# Genotype–environment associations across spatial scales reveal the importance of putative adaptive genetic variation in divergence

**DOI:** 10.1111/eva.13444

**Published:** 2022-08-24

**Authors:** Allison H. Alvarado, Christen M. Bossu, Ryan J. Harrigan, Rachael A. Bay, Allison R. P. Nelson, Thomas B. Smith, Kristen C. Ruegg

**Affiliations:** ^1^ Biology Department California State University Channel Islands Camarillo California USA; ^2^ Center for Tropical Research, Institute of Environment and Sustainability University of California Los Angeles California USA; ^3^ Department of Biology Colorado State University Fort Collins Colorado USA; ^4^ Department of Evolution and Ecology University of California, Davis Davis California USA; ^5^ Gold Country Avian Studies Nevada City California USA; ^6^ Department of Ecology and Evolutionary Biology University of California, Los Angeles Los Angeles California USA

**Keywords:** *Catharus guttatus*, cline analysis, genoscape map, gradient forest, hybrid zone, landscape genomics

## Abstract

Identifying areas of high evolutionary potential is a judicious strategy for developing conservation priorities in the face of environmental change. For wide‐ranging species occupying heterogeneous environments, the evolutionary forces that shape distinct populations can vary spatially. Here, we investigate patterns of genomic variation and genotype–environment associations in the hermit thrush (*Catharus guttatus*), a North American songbird, at broad (across the breeding range) and narrow spatial scales (at a hybrid zone). We begin by building a genoscape or map of genetic variation across the breeding range and find five distinct genetic clusters within the species, with the greatest variation occurring in the western portion of the range. Genotype–environment association analyses indicate higher allelic turnover in the west than in the east, with measures of temperature surfacing as key predictors of putative adaptive genomic variation rangewide. Since broad patterns detected across a species' range represent the aggregate of many locally adapted populations, we investigate whether our broadscale analysis is consistent with a finer scale analysis. We find that top rangewide temperature‐associated loci vary in their clinal patterns (e.g., steep clines vs. fixed allele frequencies) across a hybrid zone in British Columbia, suggesting that the environmental predictors and the associated candidate loci identified in the rangewide analysis are of variable importance in this particular region. However, two candidate loci exhibit strong concordance with the temperature gradient in British Columbia, suggesting a potential role for temperature‐related barriers to gene flow and/or temperature‐driven ecological selection in maintaining putative local adaptation. This study demonstrates how patterns identified at the broad (macrogeographic) scale can be validated by investigating genotype–environment correlations at the local (microgeographic) scale. Furthermore, our results highlight the importance of considering the spatial distribution of putative adaptive variation when assessing population‐level sensitivity to climate change and other stressors.

## 1 INTRODUCTION

Conservation strategies aimed toward preserving a species' evolutionary potential and its ability to adapt to changing environments requires an understanding of the ecological and evolutionary processes that shape intraspecific genetic variation (Morgan et al., 2020; Smith et al., 2021; Thomassen et al., 2011; Zhen et al., 2017). The field of landscape genomics is rapidly advancing as methods for generating genome‐wide datasets and detecting loci associated with adaptive divergence are applied to nonmodel organisms (Balkenhol et al., 2017; Hohenlohe et al., 2021; Waldvogel et al., 2020). In particular, understanding the spatial distribution of such adaptive genetic variation, and the underlying processes responsible for it, is critical for anticipating how species might respond to future environmental change and for developing management priorities that help mitigate negative outcomes (Allendorf, 2017; Williams et al., 2008).

One approach for understanding patterns of adaptation across a landscape is through tests of genotype–environment associations (Hoban et al., 2016), which have been studied in an array of taxa (Bennett et al., 2021; Frachon et al., 2018; Hofmeister et al., 2021; Jaffé et al., 2019; Waterhouse et al., 2018). Recent continent‐wide studies of migratory birds have identified populations most vulnerable to future climate change based on spatial variation in climate‐associated loci (Bay et al., 2018; Ruegg et al., 2018). Environmental factors, such as temperature in willow flycatchers (*Empidonax traillii*) and precipitation in yellow warblers (*Setophaga petechia*), are important predictors of putative adaptive variation. In these species, candidate loci associated with temperature and precipitation are located near functional genes that may play a role in thermal tolerance and heat stress (Ruegg et al., 2018) and migration and dispersal (Bay et al., 2018), respectively.

Although genotype–environment association analyses are a powerful tool, they inherently represent a broad brush picture across many populations (Hoban et al., 2016). Most landscape genomic studies use these broadscale patterns to infer what might be driving local adaptation (Rellstab et al., 2015), but they do not investigate whether patterns identified by broadscale analysis can be validated at the local scale. Often landscape genomic studies may be prone to false positives (De Mita et al., 2013; Frichot & François, 2015) and/or identify environmental predictors and candidate loci that are associated with some parts of the range but not others (Poncet et al., 2010). The overarching generalized patterns identified at the broad scale, however, can be further evaluated by close examination locally (Vines et al., 2016). For example, by studying a hybrid zone in one area, it is possible to identify which, if any, of the important rangewide predictors are driving local adaptation in that particular region (Hewitt, 1988, 2004). Since hybrid zone analyses can elucidate mechanisms underlying local adaptation (Rundle & Nosil, 2005), they can provide a framework at the local scale for substantiating the broadscale patterns identified by genotype–environment associations.

Here, we compare patterns of genetic variation across two different spatial scales: rangewide (macrogeographic) and local (microgeographic). We focus on hermit thrushes, *Catharus guttatus* (Pallas, 1811), a migratory songbird well‐suited to landscape genomic approaches because they occupy heterogeneous environments throughout their continent‐wide breeding range in North America. At the macrogeographic scale (i.e., across the breeding range), this species exhibits high subspecific variation in fitness‐related phenotypic traits such as morphological variation in body size, wing and bill shape, plumage coloration, and song (Aldrich, 1968; Nelson et al., 2021; Roach & Phillmore, 2017). There are three generally accepted groups of subspecies (Figure S1), and distributed among them are 12 individual subspecies (Aldrich, 1968; Dellinger et al., 2020). Two of the three groups occur in western North America, representing the majority of subspecies (10 of 12) and highest phenotypic diversity. In contrast, the third group, which contains only two of 12 subspecies, occupies the expansive area from central British Columbia (CAN) to the East coast (Dellinger et al., 2020).

At the macrogeographic scale, our objective is to investigate how genetic differentiation changes with the environment. The pattern of intraspecific diversity in hermit thrushes mirrors that of beta species diversity in birds of North America, where it is highest along the Pacific region and lowest throughout the environmentally uniform boreal areas that stretch across Canada (McKnight et al., 2007; Melo et al., 2009). At the landscape scale, habitat selection (based on elevational range) and climate adaptation (based on variation in evapotranspiration) have been shown to drive these patterns of avian beta species diversity within ecoregions of North America (Veech & Crist, 2007). Based on high phenotypic diversity of hermit thrushes throughout their western breeding range (Dellinger et al., 2020), we predict that environmentally associated genetic variation will be greatest in the west.

At the microgeographic scale, our objective is to determine whether environmental predictors indicated by the rangewide macrogeographic scale analysis can be validated at the local scale. This microgeographic analysis focuses on a hybrid zone in British Columbia, where divergent ecotypes representing two hermit thrush subspecies groups come together (Alvarado et al., 2014; Dellinger et al., 2020). This intraspecific hybrid zone between ecotypes corresponds with a migratory divide (where two populations with disparate migratory directions meet), represents a secondary contact zone between western and eastern lineages that diverged approximately 960,000 years before present (ybp) (Alvarado et al., 2014), and falls along an existing ecological gradient (Alvarado et al., 2014; Hamann & Wang, 2006).

One way to determine whether the broadscale environmental predictors are relevant to specific populations is to investigate concordance across broad and narrow spatial scales (Gugger et al., 2021). Here, we assess whether the candidate loci associated with important rangewide environmental predictors are consistent across the two scales. Concordance of clines between these loci and a local climate and/or habitat gradient would suggest a potential barrier to gene flow and/or role for ecological selection at the local scale (Vines et al., 2016). However, since broadscale genotype–environment association analyses reflect the aggregate of many local populations that may or may not differ from one another (Poncet et al., 2010), not all important environmental predictors and top candidate loci are expected to be consistent across both scales. Nevertheless, identifying which rangewide environmentally associated candidate loci also exhibit low gene flow across the hybrid zone could provide insight into ecological and evolutionary processes important to local adaptation in that region and how that may, in turn, contribute to the broadscale patterns (Rundle & Nosil, 2005; Vines et al., 2016).

To achieve our objectives, we first sequence the hermit thrush genome, generate a dataset of single‐nucleotide polymorphisms (SNPs), and conduct genome‐wide genetic analyses on samples collected at macro‐ and microgeographic scales. Specifically, for the analyses, we (i) assess rangewide genetic structure and map spatial patterns of genetic variation; (ii) identify patterns of potentially adaptive genetic variation rangewide; (iii) identify candidate loci associated with top rangewide environmental predictors; (iv) conduct cline analysis of genetic data, including the top rangewide candidate loci, at a hybrid zone to evaluate potential drivers of adaptation at the local scale; and (v) discuss the conservation implications of our findings in the context of future climate change. To accomplish this, for the rangewide dataset, we use gradient forest (Ellis et al., 2012) to identify areas of high putative adaptive genetic variation and important environmental predictors. Next, we use latent factor mixed models (Frichot et al., 2013) to identify top environmentally associated loci, after accounting for population structure. Then, at the hybrid zone, we use HZAR (hybrid zone analysis for R) (Derryberry et al., 2014) to compare clines for overall genomic variation including the top environmentally associated candidate loci identified in the rangewide analysis. Together, analyses at the micro‐ and macrogeographic scales can improve our understanding of the environmental drivers of genetic variation, including how the rangewide patterns can be validated through investigations of putative adaptation at the local scale.

## 2 MATERIALS AND METHODS

### 2.1 Sampling and DNA extraction

**FIGURE 1:**
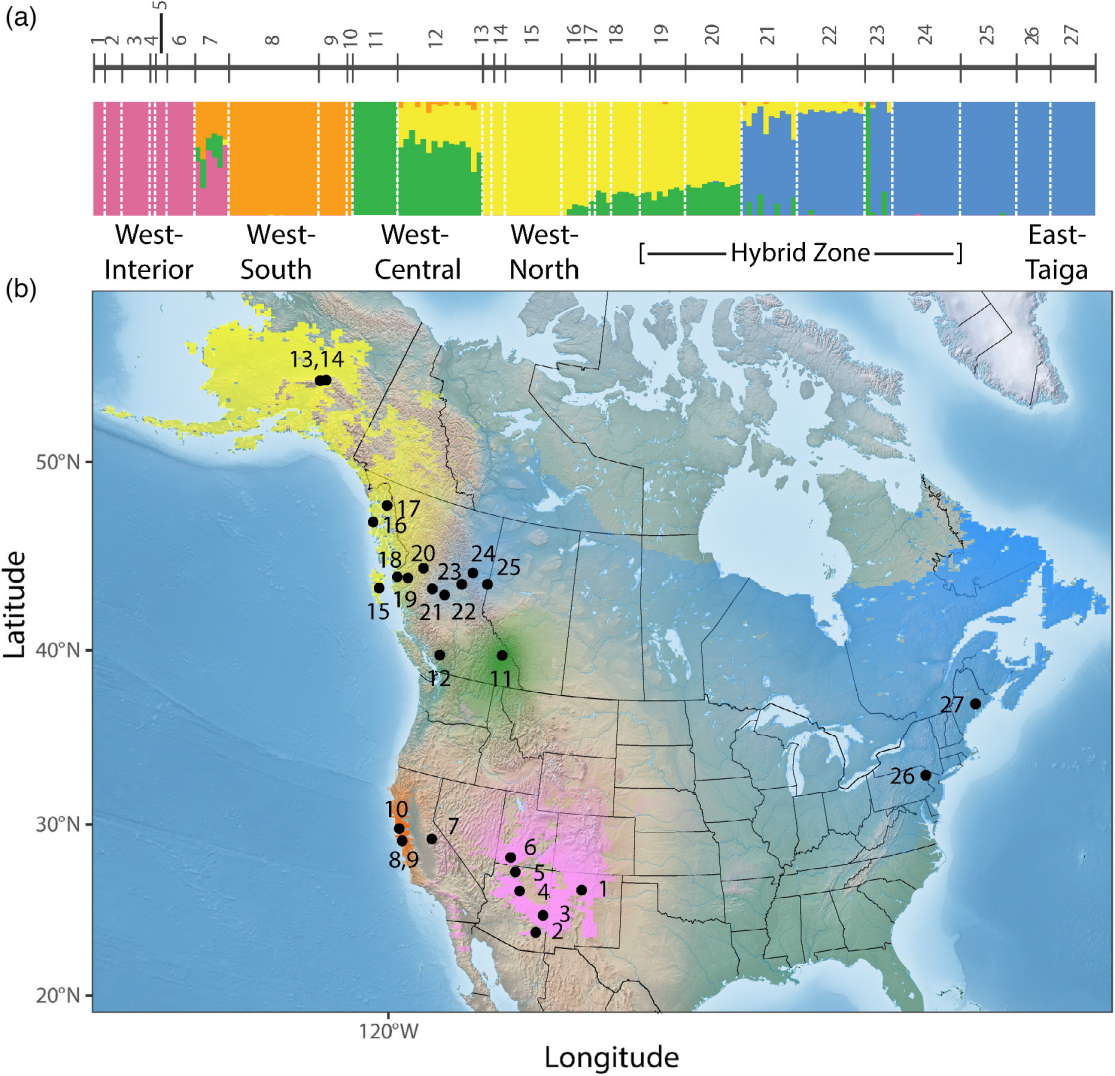
Genetic structure of populations across the hermit thrush breeding range, demonstrating high genetic structure in western North America and limited genetic structure throughout the boreal and eastern regions. (a) Results from ADMIXTURE illustrating five genetically distinct populations, including cluster names, across the breeding range for the full genomic dataset of 90,439 SNPs. Numbers refer to breeding site locations depicted on the map in panel b and are identified in Table 1. (b) Spatially explicit map of population genetic structure across the breeding range. The colors correspond to the five genetic clusters (*K* = 5). The density of each color reflects the posterior probability of membership for each pixel to the most probable of the five genetic clusters. Transparent color appears on the map in areas of admixture (i.e., mixed posterior probability and thus uncertain assignment). Due to admixture among the four western clusters, the relatively continuous distribution (see Figure 2a) of hermit thrushes throughout the west is not apparent on this map.

Blood samples of 310 individuals were obtained from across the breeding range of the hermit thrush (Figure 1). Samples were collected from birds during the breeding season (May through August). Individual samples were grouped into sites, defined as individuals breeding within one degree latitude and longitude with no more than 10% difference in any environmental variable (as indicated by our environmental predictors). This resulted in 27 sites across the breeding range (Figure 1). In Table 1, each sited is labeled according to its subspecies group, for which the range limits tend to be better defined compared with those of the subspecies (Dellinger et al., 2020). Our rangewide sampling scheme includes 13, 7, and 7 sites from within the range of Western Lowland, Western Mountain, and Northern subspecies groups, respectively (Table 1, Figure S1). This is proportional to the number of subspecies and phenotypic variation within each group (i.e., sampling was heavily focused across the regions of higher phenotypic variation where genetic differences were more likely to be pronounced). For the microgeographic analysis, we focus on a hybrid zone where the Western Lowland and Northern subspecies groups, reflecting the western and eastern lineages (Alvarado et al., 2014), meet in British Columbia. This represents the best known split between the main subspecies groups, and as a result, there is thorough sampling from this area across a known environmental gradient. From all samples, DNA was extracted using the Qiagen™ DNeasy Blood and Tissue extraction kit according to the manufacturer’s protocols and quantified using the Qubit® dsDNA HS Assay kit (Thermo Fisher Scientific).

**TABLE 1:** Sampling sites across the hermit thrush breeding range

Site	Group	State	Site name	*N*	Latitude	Longitude
1	WM	NM	Los Alamos	2	35.84	−106.42
2	WM	AZ	Pinaleno Mtn	3	32.67	−109.88
3^a^ ^,^ ^b^	WM	AZ	Big Lake	5	33.88	−109.43
4	WM	AZ	Flagstaff	1	35.34	−111.6
5	WM	AZ	Jacob Lake	2	36.6	−112.18
6^a^ ^,^ ^b^	WM	UT	Routes 14/18	5	37.53	−112.75
7^a^ ^,^ ^b^	WM	CA	Yosemite	6	37.77941	−119.7566033
8^a^ ^,^ ^b^	WL	CA	Big Basin site 1	16	37.175385	−122.21825
9^a^ ^,^ ^b^	WL	CA	Big Basin site 2	5	37.166457	−122.20749
10	WL	CA	Bolinas	1	37.933518	−122.727446
11^a^ ^,^ ^b^	WL	BC	Golden	8	51.06567125	−116.8025
12^a^ ^,^ ^b^	WL	BC	Whistler	15	50.12456	−123.2933333
13	WL	AK	Hwy Pass/Toklat	2	63.49411	−150.09
14	WL	AK	Mile17	2	63.73674	−149.372
15^a^ ^,^ ^b^	WL	BC	Haida Gwaii	10	53.162545	−131.8095
16^a^ ^,^ ^b^	WL	AK	Sitka	5	57.0738956	−135.3395108
17	WL	AK	Juneau	1	58.42	−134.55
18^a^ ^,^ ^b^ ^,^ ^c^	WL	BC	Prince Rupert	8	54.27972625	−130.33425
19^a^ ^,^ ^c^	WL	BC	Exstew	8	54.44668872	−129.1349303
20^a^ ^,^ ^c^	WL	BC	Kispiox	10	55.397947	−127.8039
21^a^ ^,^ ^c^	N	BC	Maxan	10	54.26883	−126.1004
22^a^ ^,^ ^c^	N	BC	Fort Fraser	12	54.10814445	−124.569797
23^a^ ^,^ ^c^	N	BC	MacKenzie	5	55.10428	−122.9972
24^a^ ^,^ ^c^	N	BC	Hudson's Hope	12	56.01897	−122.0631667
25^a^ ^,^ ^b^ ^,^ ^c^	N	BC	Swan	10	55.516232	−120.0838
26^a^ ^,^ ^b^	N	PA	Portertown	6	41.27183433	−75.177813
27^a^ ^,^ ^b^	N	ME	Penobscot	8	44.846	−68.616

*Note*: Subspecies group: Western Mountain (WM), Western Lowland (WL), or Northern (N).

Sites 1–27 are used in analysis of population structure. Additional symbols denote:

^a^ Subset of 19 sites used for *F*_ST_ and partial Mantel tests.

^b^ Subset of 13 sites used in the gradient forest and latent factor mixed models.

^c^ Subset of 8 sites used in the cline analysis.

### 2.2 Genome assembly

Blood from a single hermit thrush individual (band number 2471–25909) from Sitka, Alaska was collected and sent to the University of California Davis (UC Davis) Genome Center for high molecular weight DNA isolation and 10× Genomics Chromium Genome library preparation. The 10× method uses microfluidics to separately partition and barcode smaller portions of the genome, information which can be leveraged for larger‐scale assembly and phasing. Sequencing of the 10× library was performed as PE150 on a half lane (to target 60× coverage as suggested by 10×) of an Illumina Hiseq X Ten run (Illumina). To assemble the hermit thrush genome, we implemented the workflow provided with the Supernova assembler v2.1.1 (10× Genomics, San Francisco, CA, USA) (Weisenfeld et al., 2017). We first ran the mkfastq script, which demultiplexes the Illumina sequencer's base call files (BCL). These paired‐end reads were input to the *supernova run* module for de novo assembly. We used default parameter values, with the exception of no ‐maxreads argument so that we used all reads for assembly. To remove potential minor contents of sequence cross‐contamination, which occurs when libraries are multiplexed in a sequencing lane (Costello et al., 2018), we ran a decontamination workflow using custom scripts to identify and remove sequences that are misassigned. First, we filtered the barcodes and generated raw reads after barcodes were cut off. Next, we created a *bam* file by mapping the FASTQ files produced above to the original genome assembly using *bwa* (Li & Durbin, 2009). Finally, we sorted *bam* files to calculate and plot the median ratio (i.e., the number of reads in the genome barcode/ number of reads mapped to the scaffold) compared with length of scaffold. We defined potential contaminants as scaffolds less than 1 kb that had a median ratio less than 10, and we removed 39 such scaffolds from the genome assembly.

### 2.3 SNP discovery and SNP filtering

We performed genome scans on 310 individuals following bestRAD library preparation protocol with some modifications (Ali et al., 2016). In short, DNA was normalized to a final concentration of 100 ng in a 10 μl volume and digested with restriction enzyme SBfl (New England Biolabs, NEB). The fragmented DNA was then ligated with SBfI specific adapters prepared with biotinylated ends, and samples were pooled and cleaned using 1× Agencourt® AMPure XP beads (Beckman Coulter). Pooled and clean libraries were sheared to an average length of 400 bp with 10 cycles on the Bioruptor NGS sonicator (Diagenode) to ensure appropriate length for sequencing, and an Illumina NEBNext Ultra DNA Library Prep Kit (NEB) was used to repair blunt ends and ligate on NEBNext Adaptors to the resulting DNA fragments. Agencourt® AMPure XP beads (Beckman Coulter) were then used to select DNA fragments with an average length of 500 bp, libraries were enriched with PCR, and cleaned again with Agencourt® AMPure XP beads. The resulting libraries were sequenced on four lanes of an Illumina HiSeq 2500 at the UC Davis Genome Center using 250 base pair, paired‐end sequencing. The final two lanes included 124 individuals with low coverage from the first two sequencing lanes; thus, these libraries were resequenced along with an additional 68 new individuals.

We used the program *Stacks v2.60* (Catchen et al., 2013) to demultiplex, filter and trim adapters from the data with the *process_radtags* function. Duplicate read pairs were removed using the *clone_filter* function in *Stacks*. Only cases in which both reads in a pair passed quality filters were used in downstream analysis. Reads were mapped to our genome assembly using *bowtie2* (Langmead & Salzberg, 2012). We then used the Haplotype Caller in the Genome Analysis Toolkit to detect single‐nucleotide polymorphisms (SNPs), following best practices from the Broad Institute (http://www.broadinstitute.org). In an initial round of variant filtration, we discarded low‐quality variants (genotype quality < 30; depth < 8; minor allele frequency < 0.03), as well as indels and nonbiallelic SNPs using *vcftools* (Danecek et al., 2011). Using this quality filtered set, we conducted a second round of filtration by visualizing the trade‐off between discarding SNPs with low coverage and discarding individuals with missing genotypes to determine which SNPs and individuals to discard in the R software program *genoscapeRtools* (Anderson, 2019).

### 2.4 Population structure and genoscape map

To assess population genetic structure across the hermit thrush breeding region, we used the software program ADMIXTURE (Alexander et al., 2009), a maximum likelihood model‐based approach to estimate ancestry of 178 individuals (Table 1). The model was run with a burn‐in period of 50,000, and a total run length of 150,000 generations. We ran five iterations of each assumed number of genetic clusters (*K*), where *K* ranged from 1:7 (Figure S2). We used the R software program *pophelper* (Francis, 2017) to visualize each run, as well as to estimate the cross‐validation error to determine the optimal *K*.

To create the genoscape, a spatially explicit map of genetic clustering, we visualized the posterior probability of group membership estimates from ADMIXTURE as transparency levels of different colors overlaid on a base map from Natural Earth (https://www.naturalearthdata.com/) and clipped this to a map of the hermit thrush breeding range (NatureServe, 2018). We scaled the transparency of colors within each distinguishable group, so that the highest posterior probability of membership in the group according to structure was opaque and the smallest was transparent.

### 2.5 Association between environmental predictors and genomic data

To determine whether changes in genetic allele frequencies were associated with particular environmental variables, we extracted a suite of 25 climate, vegetation, and anthropogenic characteristics from 19 sampling locations. For climate predictors, we used the 19 bioclimatic layers available as part of the WorldClim 2.1 database (Hijmans et al., 2005), which captures temperature and precipitation measurements averaged across the time period 1970–2000. Data from these layers was extracted at 30‐arc second resolution (~1km^2^). We included three layers representing vegetation and characteristics: (1) two measures of the normalized difference vegetation index (NDVI) captured by MODIS instrument (Carroll et al., 2004) that represented the maximum and standard deviation of NDVI at each location for the year 2003 (chosen as a representation of the vegetation seen at each location), and (2) a layer capturing tree cover in each habitat (Sexton et al., 2013) at ~1km^2^ resolution. We used a measure of surface moisture estimated by the NASA Scatterometer Climate Record Pathfinder (QuickSCAT) representing the mean of surface moisture available at each site. We included the elevation at each site as a predictor, as captured by the Shuttle Radar Topography Mission (SRTM, also at 30‐arc second resolution and downloaded via the worldclim.org website). Finally, we included an amalgamated measure of anthropogenic activity, the human influence index (HII), that represents nine independently collected anthropogenic activities, such as population density, construction density and land use, nighttime lights, and access to locations (road and rail density, coastlines) (WCS, 2005). Latitude and longitude were also included in the analysis as potential predictors and to tease apart any effects of geography (Section 2.7).

To link the potential environmental predictors to our genomic data at the macrogeographic scale, we used the package *gradientForest* (Ellis et al., 2012) in R (R Core Team, 2017). For the initial gradient forest analysis, we ran the gradient forest using all 19 populations that had at least five individuals (Table 1). However, for the final gradient forest analysis, we included 13 populations and excluded six sites from the hybrid zone. Gradient forest modeling examines the overall spatial distribution of genomic variation across a landscape (Bay et al., 2018; Fitzpatrick & Keller, 2015). Gradient forests are rooted in random forest models (Breiman, 2001), and attempt to link multiple responses (in this case, allelic variation across the genome between populations) to predictor variables (in this case, the above‐described environmental characteristics of each population) using a bifurcating, iterative, nonparametric regression algorithm. The results of these regressions are an estimate of the number of responses with significant associations with predictor variables, an estimate of the strength of such associations, and a ranking of the predictor variables relative to one another across all responses tested. An advantage of these models is that they are iterative in nature, and because they withhold both records and predictor variables randomly with each iteration, they are largely immune to biases due to multicollinearity and/or spurious correlations. We compared these observed results with those obtained from gradient forests that were run with randomized matches between genomic and environmental records (*n* = 50), to estimate the difference between our observed associations between environment and genomic data as compared with randomized ones.

### 2.6 Predicting genomic turnover across the breeding range

To create a spatially explicit visualization illustrating how genomic variation changes with environmental conditions across geographic and environmental space, we generated a map and PC plot as follows. We used the estimated relationship between genomic data and environment of the hermit thrush, as revealed by gradient forests, to predict the changes in genomic variation across the entire range of the species. Due to the fact that this variation is based on loci linked to environment variables, we refer to these changes as putatively adaptive allelic turnover. We estimated this putatively adaptive allelic turnover by randomly selecting 10,000 geographic points across the range of the hermit thrush and extracting the values of the three environmental variables most strongly correlated with genomic variation, as determined by the gradient forest model run on the 13 sites. We then predicted the genomic variation at each of these sites based on the relationship between genomics and these environmental variables obtained from gradient forest model run on the 13 sites. We then used ordinary kriging to interpolate between unsampled locations across the breeding range (Oliver & Webster, 1990).

### 2.7 Association between geographic distance, environmental distance, and genomic data

To assess the relationship between geographic, environmental, and genetic distances, we also calculated pairwise *F*_ST_ across all quality filtered SNPs using the R package *assigner* version 0.5.6 (Gosselin et al., 2019). Here, we used the hierfstat model (Goudet, 2005) to also provide confidence intervals surrounding the *F*_ST_ estimates. For *F*_ST_ and subsequent analyses incorporating environmental and geographic distance (Table 1), we included all 19 populations that had at least 5 individuals. Geographic distance was calculated using the package *geosphere* (Hijmans, 2019) which calculates the distances using the Vincenty inverse formula for ellipsoids to account for the curve of the earth. Environmental distance between sites was calculated as the Euclidean distances based on the top three environmental variables identified in gradient forest (mean diurnal temperature range (BIO2), temperature seasonality (BIO4), and annual temperature range (BIO7)) using the function *dist* in R (R Core Team, 2019).

To determine the influence of geographic distance versus environmental distance on patterns of genomic variation across the hermit thrush breeding range, we performed several tests. First, we included the mean latitude and longitude of each population as predictors in the gradient forest runs (for a total of 27 potential predictors, Table S1), allowing the influence of geography to compete directly with environmental predictors at each site. Second, to create a visual comparison of the relative influence of geography vs environment on genetic distances, we calculated, within each genetic cluster (based on population structure analysis described in Section 2.4), the relative mean within‐group Euclidean distance for geography versus the relative mean within‐group Euclidean distance for environment. Third, we performed partial Mantel tests between *F*_ST_, geographic distance, and environmental distance using R package *vegan* (Oksanen et al., 2015) for the 19 populations. In addition, to test whether isolation by environment is more important than isolation by distance in the western lineage compared with the eastern lineage, we ran the partial Mantel tests for the two lineages separately.

### 2.8 Identifying candidate loci for top climatic variables

At the macrogeographic scale, to identify candidate variants that were strongly associated with the rangewide environmental predictors, we ran latent factor mixed models (LFMM) (Frichot et al., 2013) for the same 13 sites included in the final gradient forest analysis (i.e., this excluded six sites from the hybrid zone) (Table 1). These models are statistical regression models to test associations between a multidimensional set of response variables (i.e., genotypes) and a set of variables of interest (i.e., environmental variables), while correcting the model for confounding effects such as population structure. Here, we tested for correlations between all quality filtered SNPs and the top‐ranking uncorrelated environmental variables identified in the gradient forest analyses (mean diurnal temperature range (BIO2), temperature seasonality (BIO4), and maximum temperature of the warmest month (BIO5)) whose Pearson's correlation coefficient was 0.75 or less. For each of the environmental variables from the gradient forest analysis, we ran five separate MCMC runs using the Bayesian *LFMM version 1.5* (Frichot et al., 2013) with a latent factor of *K* = 5, based on an initial PCA to identify underlying genetic structure with the *prcomp* function in the *stats* v3.6.2 package in R (R Core Team, 2020). *p*‐Values from all five runs were combined and adjusted for multiple tests using a false discovery rate (FDR) correction.

To compare candidate loci associations across geographic scales, we additionally ran LFMM for mean diurnal temperature range (BIO2), temperature seasonality (BIO4), and maximum temperature of the warmest month (BIO5) (i.e., top uncorrelated environmental variables from macrogeographic scale) for the populations within the hybrid zone using the same parameters used for the previous analysis. This identified the top 10 candidate loci for mean diurnal temperature range (BIO2), temperature seasonality (BIO4), and maximum temperature of the warmest month (BIO5) at the microgeographic scale and their association within the hybrid zone. Across the eight hybrid zone sites in British Columbia (Table 1), we mapped allele frequencies of the top rangewide candidate locus associated with each of the top three uncorrelated environmental variables, which was overlaid on a background showing changes in the associated bioclimatic variable across the hybrid zone.

For candidate loci found to be highly ranked at both macro‐ and microgeographic scales, we subsequently investigated the closest genic region. To do this, we used the Satsuma synteny program (Grabherr et al., 2010) to align the hermit thrush genome to the Zebra Finch chromosomal genome assembly, converting the scaffold position to a chromosomal position with an associated annotation. We used NCBI blast by generating DNA sequence segments that include 200 bp surrounding each candidate loci and blasting the segment against the Zebra Finch genome. We also extended our exploration to genes within 25 kb upstream or downstream of our top candidate variants, which we assume is within the distances before which LD should break down (Backström et al., 2006).

### 2.9 Cline analysis

We used the program HZAR (hybrid zone analysis for R) (Derryberry et al., 2014), implemented in the R programming environment (R Core Team, 2019), to fit all clines and perform all clinal analyses across the eight hybrid zone sampling locations in British Columbia (Table 1). First, we fit the best of three models (as determined with AIC comparisons) to the ancestry proportions calculated in ADMIXTURE (*K* = 2), as well as the population‐scaled and transformed environmental PC values and each variable separately. Each model was used to predict cline parameters (center and width). In addition to the null model, the models used for the cline fitting were as follows: model I: no exponential tail is desired; model II: a model where just one exponential tail on the right is desired; and model III: where two exponential tails mirrored about the cline center is desired. We ran each model on three chains, for 1 × 10^6^ generations with a 10% burn‐in period. We assessed convergence using trace plots and once the best model had been selected, it was used to plot the best fit cline for the observed data, as well as the confidence interval around the cline. We additionally looked at the cline of allele frequencies of top candidate loci identified in the rangewide LFMM analyses.

## 3 RESULTS

### 3.1 Genome assembly, SNP discovery, and SNP filtering

The final size of the assembled genome was 1.034 Gb, comparable to the average size of bird genomes, which is remarkably conserved across avian taxa (Gregory, 2005; Zhang et al., 2014). The scaffold N50 was 35.7 Mb and the longest scaffold was 105.36 Mb. GC‐content of the genome was 41.86%. The scaffold N90 was 2.23 Mb; 90% of the total length of the assembly lies in 40 scaffolds greater than this length.

In total, we identified 4,703,497 variants across the genome. We filtered out variants with greater than 10% missing genotypes and variants with a minor allele frequency less than 3%. We additionally discarded low coverage individuals missing more than 25% of SNPs for a final set of 90,439 SNPs and 178 individuals (Table 1).

### 3.2 Population structure

We identified five genetic clusters across the breeding range (Figure 1 and Table 1). The genomic breaks in the ADMIXTURE plot at *K* = 3 (Figure S2) are consistent with the three main subspecies groupings. At *K* = 5, the Western Lowland subspecies group is further subdivided based on additional genomic breaks within this group (Figure 1). The blue genetic cluster, which we refer to as East‐Taiga, spans from the East coast across Canada to central British Columbia (sites 24–27 have little to no admixture) and corresponds to the Northern subspecies group. The pink genetic cluster, which we refer to as West‐Interior, occupies the Madrean and Rocky Mountain ranges (sites 1–6 have no admixture) and corresponds to the Western Mountain subspecies group. The remaining three genetic clusters occur along the West coast, where they segregate by latitude. In aggregate, they correspond to the Western Lowland subspecies group. The West‐North cluster (yellow) is found in Alaska and northwestern British Columbia (sites 13–15 have no admixture). The West‐Central cluster (green) is found in southern British Columbia (site 11 has no admixture). Finally, the West‐South cluster (orange) is found in California (sites 8–10 have no admixture). There are several locations across the range with admixture (Figure 1 and Table 1), including site 7 in Yosemite, CA, sites 16–17 in southeast Alaska, and sites 12 and 18–23 in British Columbia.

### 3.3 Association between geography, environment, and genomic data

When we analyze all the western sites together, the contrasting patterns between the eastern and western lineages emerge. Across all sites, we find a wide range of *F*_ST_ values (0.0003–0.1788). As expected, the highest values of *F*_ST_ occur between the eastern and western lineages (Table S2). When the two lineages are analyzed separately, we observe relatively low *F*_ST_ values within the eastern lineage and high *F*_ST_ values within the western lineage (including for pairwise comparisons among sites with similar geographic distances, for example, site 24 to 27 vs. site 3 to 17) (Figure 1 and Table S2). Partial Mantel tests consistently reveal contrasting patterns for eastern and western lineages when analyzed separately (Table S3). The western lineage shows significant patterns of isolation by environment (*r* = 0.414, *p* = 0.01) and isolation by distance (*r* = 0.657, *p* = 0.001). In contrast, the eastern lineage shows no evidence of isolation by environment (*r* = 0.189, *p* = 0.176) or isolation by distance (*r* = 0.181, *p* = 0.114).

### 3.4 Association between environmental predictors and genomic data

We found a strong relationship between environmental variables and genomic variation in our rangewide analysis of hermit thrushes. Important predictors in our 13 site model were BIO2 = mean diurnal temperature range (mean of monthly [max temp – min temp]), BIO4 = temperature seasonality (standard deviation × 100), and BIO7 = annual temperature range (max temp of warmest month – min temp of coldest month) (Table S1). Each of these variables explained a larger portion of genomic variation than latitude or longitude (Table S1) and suggest a stronger association between environment and genomic variation than could be explained by geography alone. Of the 90,439 loci that were included in the gradient forest analysis, over 47% (*n* = 42,561) were able to be explained by available predictors (e.g., *R* ^2^ greater than 0), and these responses had an average correlation of *R* ^2^ = 0.26 with predictor variables. These responses were significantly greater in number and correlation value than those gradient forests where genomic signatures and predictors were randomized with respect to one another (*p* < 0.01, Figure S3). Even when considering predictors that are not highly correlated (Pearson's correlation coefficient <0.75) (Table S4), the top two predictors (BIO2 = mean diurnal temperature range, BIO4 = temperature seasonality) remain the same, and the third ranked variable is replaced by another temperature metric (BIO5 = maximum temperature of the warmest month).

### 3.5 Predicting genomic turnover across the breeding range

**FIGURE 2:**
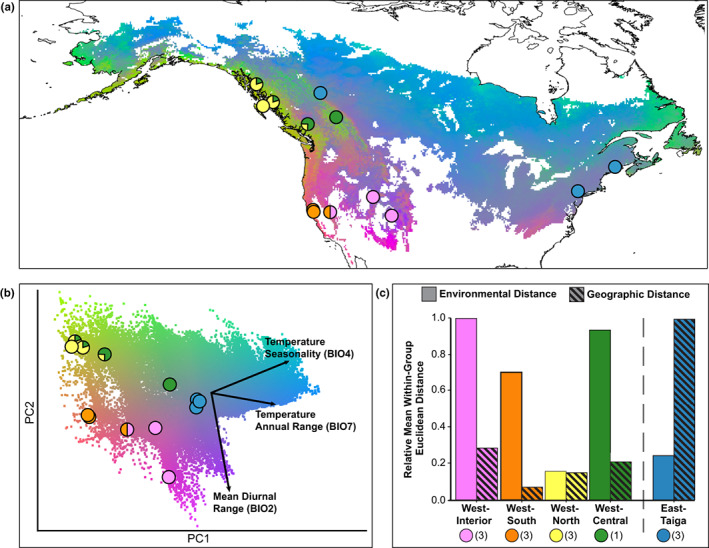
Genotype–environment associations across the hermit thrush breeding range, indicating relatively high turnover of putatively adaptive alleles in western North America. (a) Gradient forest‐based genomic signatures mapped to geography support climate adaptation across the breeding range and higher turnover of putatively adaptive allelic variation in the western region compared to the boreal and eastern regions. Background colors on map are based on modeled gene–environment correlations predicted at 100,000 random points across the breeding range. Circles on map represent sampling locations and are colored according to the corresponding genetic cluster from Figure 1. (b) Principal component analysis of gradient forest predictions of genomic signature. Background color represents environmental space, whereas circles are positioned to reflect PC scores associated with each sampling location (colored according to genetic cluster). The western clusters are separated throughout environmental space, whereas sites associated with the East‐Taiga cluster are tightly grouped together. (c) Plot of relative mean within‐group Euclidean distances (Environmental vs Geographic) for each genetic cluster reveals contrasting patterns. Each western cluster shows high environmental distances across relatively small geographic distances, whereas the East‐Taiga cluster shows low environmental distance across large geographic distances. Numbers in parentheses represent within‐cluster pairwise comparisons.

Gradient forest analysis also allowed for visualization of environmentally associated allelic variation at the broad spatial scale (Figure 2a), revealing strong differences across the breeding range. Changes in background color on the map represent turnover in the relationship between environmental variables and these putatively adaptive alleles. The map shows high variation in genotype–environment associations (i.e., high turnover) across relatively small geographic areas throughout the west; this contrasts the low variation in genotype–environment associations (i.e., low turnover of alleles) across the large geographic area spanning from central British Columbia to the East coast (Figure 2a). On the map, circles indicate the sampling sites and are color coded to represent the genetic cluster associated with the corresponding site on the genoscape map (Figure 1).

The PC plot (Figure 2b) represents environmental space and includes vectors for important environmental predictors (mean diurnal temperature range (BIO2), temperature seasonality (BIO4), and annual temperature range (BIO7)). These variables are all related to temperature. Thus, the pattern is predicted by climate variables generally, and specifically with respect to temperature. The top three uncorrelated environmental predictors are all related to temperature as well. BIO2 and BIO4 stay the same, and BIO5 replaces BIO7 (as BIO4 and BIO7 are correlated) (Table S2). We keep both BIO4 and BIO7 here as gradient forest models are largely immune to collinearity; however, we consider only the uncorrelated variables when we selected variables to explore as part of rangewide LFMM and analyses for the hybrid zone (Section 3.6).

For visualization purposes, the PC plot is oriented such that the background color representing environmental space (Figure 2b) corresponds spatially to the background color representing environmentally associated allelic variation on the map (Figure 2a). The PC plot indicates where the 13 sampling sites are distributed in environmental space relative to one another (i.e., separating out or grouping together), and each site is colored according to its genetic cluster in Figure 1. Sites associated with the East‐Taiga genetic cluster (blue circles) are grouped tightly within the PC plot (Figure 2b). This indicates very little environmental variation between East‐Taiga sites despite large pairwise geographic distances between some of them (Figure 2a,c), thus substantiating the low variation in genotype–environment associations across this area. In contrast, within the western region, sites associated with each genetic cluster (pink, orange, yellow, and green circles) are dispersed throughout the PC plot (Figure 2b). This indicates relatively high environmental variation (i.e., high environmental heterogeneity) between sites despite shorter geographic distances within each cluster (Figure 2a,c), thus substantiating the high variation in genotype–environment associations across the west. The bar chart (Figure 2c) provides a side‐by‐side comparison of sites grouped according to each of the five genetic clusters from the genoscape (Figure 1). For the East‐Taiga cluster, environmental distance is low relative to large geographic distances. The opposite pattern exists for most of the western clusters, where environmental distance is high compared with relatively small geographic distances.

### 3.6 Identification of candidate loci for top climatic variables

To investigate genomic loci potentially involved in climate adaptation of hermit thrush populations, we used LFMM (Frichot et al., 2013) to identify 2848 genomic loci associated with the top three uncorrelated climatic variables ranked in the rangewide gradient forest analyses described above, which excluded six of the hybrid zone populations. We identified 2138 candidate variants associated with mean diurnal temperature range (BIO2), 1112 associated with temperature seasonality (BIO4), and 1375 associated with maximum temperature of the warmest month (BIO5). We identified the top 10 variants associated with each of the top three uncorrelated climatic predictors on the macrogeographic scale (Figure S4), and we determined that these loci varied in their importance within the hybrid zone (i.e., ranking of association across the hybrid zone) (Figure S5). No variants associated with mean diurnal temperature range (BIO2) or maximum temperature of the warmest month (BIO5) overlap as a highly ranked candidate locus at both scales. However, two variants rose to the top at both scales, and they were both associated with temperature seasonality (BIO4).

### 3.7 Hybrid zone analysis

**FIGURE 3:**
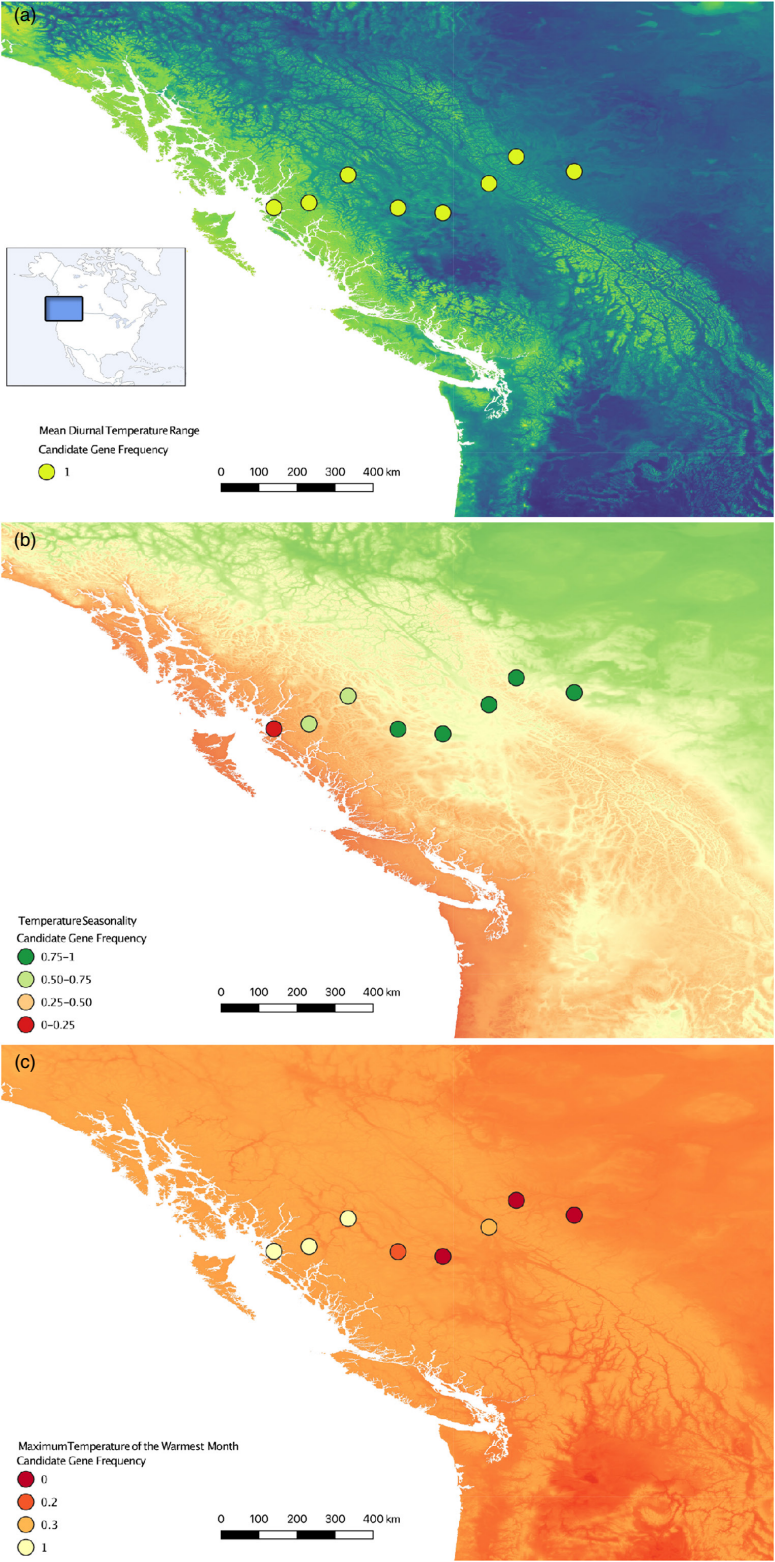
Variation in patterns of allele frequency changes across the hybrid zone in British Columbia for temperature‐associated top candidate loci identified in the rangewide analysis. (a) Allele frequency for the top candidate locus associated with mean diurnal temperature range (BIO2) is fixed. (b) Allele frequency for the top candidate locus associated with temperature seasonality (BIO4) shows a large shift close to the coast. (c) Allele frequency for the top candidate locus associated with maximum temperature of the warmest month (BIO5) shows a large shift farther inland. The color within each circle represents the frequency of the highest ranked allele (as determined by the rangewide LFMM analyses) across the eight sampling sites, while the underlying map represents the gradient across the hybrid zone of the associated bioclimatic variable.

At the microgeographic scale, when tracking the allele frequency changes across the hybrid zone, the pattern varies between the top temperature‐associated candidate loci. Figure 3 shows the allele frequency changes overlaid on a background representing the raw environmental data for mean diurnal temperature range (BIO2), temperature seasonality (BIO4), and maximum temperature of the warmest month (BIO5). The top candidate locus associated with mean diurnal temperature range (BIO2) is fixed and shows no change in allele frequency (Figure 3a), while the top candidate locus associated with maximum temperature of the warmest month (BIO5) does show an allele frequency shift across the cline (Figure 3c). The top candidate locus associated with temperature seasonality (BIO4) not only shifts in frequency across the hybrid zone but also tracks the gradient of the associated bioclimatic variable (Figure 3b). Notably, it was this locus along with the second top candidate locus (both associated with temperature seasonality (BIO4)) (Figures S4b and S5b) that overlap as important candidates at both the micro‐ and macrogeographic scales. These top two variants for temperature seasonality (BIO4) were both found in the same genomic region, and our analyses indicate this region to be on chromosome 2, upstream of the uncharacterized protein LOC105759009 and the prolactin gene, *PRL*. Thus, the changes in allele frequency of these two candidate variants (both falling near the prolactin gene, *PRL*) are closely aligned with a gradient in temperature seasonality (BIO4) across British Columbia.

**FIGURE 4:**
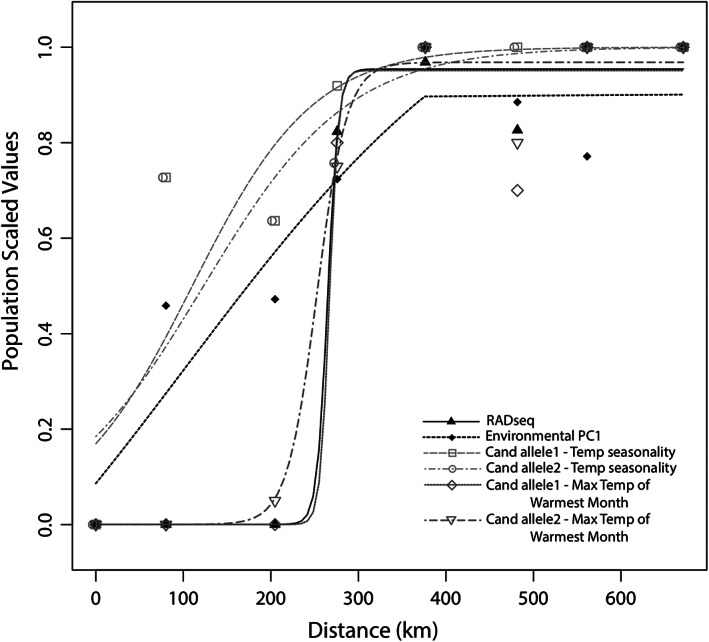
Geographic cline plots show the relationship between the genomic cline, temperature gradient, and candidate loci across the hybrid zone in British Columbia. The clines for candidate loci associated with temperature seasonality (BIO4) (gray open squares and circles) and Environmental PC1 (black diamonds) are shifted to the left of the genomic cline (RADseq; black triangles), which represents the ancestry estimates from the full genomic dataset (90,439 SNPs) with a *K* = 2. The Environmental PC1 cline (black diamonds) represents scaled top uncorrelated climatic variables (mean diurnal temperature range (BIO2), temperature seasonality (BIO4), and maximum temperature of the warmest month (BIO5)) identified by the gradient forest analysis. The clines for the candidate loci associated with maximum temperature of the warmest month (BIO5) are closely associated with the genomic cline. Candidate loci associated with mean diurnal temperature range (BIO2) are fixed and are not included here.

HZAR clines were calculated for population specific measures of RADseq ancestry, environmental space defined by scaled top rangewide uncorrelated climatic variables, and candidate loci associated with two of the top environmental predictors (as identified by LFMM in the broadscale analysis) (Figure 4). Mean diurnal temperature range (BIO2) is not included here as the allele frequencies of the top two candidate loci were fixed (Figure 3a). The ancestry cline was steep and narrow, centered at 264.9 km (range = 252.0–273.7 km) with a width of 24.3 km (range = 6.0–27.1 km). The Environmental PC1 cline was shallower and shifted left (center = 206.5 km, width = 196.2 km). The clines of the two candidate loci associated with temperature seasonality (BIO4) follow a pattern similar to the Environmental PC1 cline, whereas the clines of the two candidate loci associated with maximum temperature of the warmest month (BIO5) are more similar to the genomic cline (Figure 4).

## 4 DISCUSSION

Genotype–environment association analyses at the macrogeographic scale support the hypothesis of higher turnover of putatively adaptive alleles in the western breeding range of hermit thrushes compared with eastern and boreal breeding areas. This is consistent with known variation in fitness‐related phenotypic traits, which is also highest throughout the western region (Dellinger et al., 2020). Bioclimatic variables associated with temperature are important predictors of these broadscale patterns, and we identify loci that are associated with the top‐ranking uncorrelated temperature variables after accounting for rangewide population structure. As broadscale analyses inherently reflect a composite of many local populations and thus represent an overarching generalized pattern (Hoban et al., 2016; Rellstab et al., 2015), we investigate whether the macrogeographic pattern can be validated at the microgeographic scale. Although there is variation in the importance of rangewide predictors, our hybrid zone analysis confirms that temperature is likely an important driver of putative local adaptation in British Columbia, where we find low gene flow between ecotypes and a potential role for ecological selection driven by a temperature gradient. Here, we discuss possible ecological and evolutionary mechanisms underlying the patterns at both spatial scales, and we then address the conservation implications of geographic variation in the distribution of climate‐linked putatively adaptive genetic variation.

### 4.1 Climate‐associated genomic diversity across the breeding range

Our study supports a role for the environment in shaping intraspecific genomic diversity at the macrogeographic scale, including an uneven spatial distribution of putative adaptive genetic variation across the breeding range. In particular, high population structure (Figure 1) and high variation in genotype–environment associations throughout the west (Figure 2a,b) starkly contrasts low population structure and a lack of variation in genotype–environment associations throughout the eastern and boreal regions. Gradient forest revealed strong support for temperature‐associated genetic diversity above what was expected by chance, indicating that this pattern does not merely reflect neutral population structure (Ellis et al., 2012). LFMM further revealed candidate loci strongly associated with mean diurnal temperature range (BIO2), temperature seasonality (BIO4), and maximum temperature of the warmest month (BIO5) after accounting for population structure (Frichot et al., 2013).

The comparison between east and west in geographic and environmental distance further supports a strong role for the environment in driving patterns of genetic diversity. If geographic distance was an important factor, we would predict more divergence in the east compared to the west; however, we found the opposite. In hermit thrushes, the pattern and environmental predictors of putative adaptive genetic variation parallel those for beta diversity (i.e., high turnover) of bird species in North America (McKnight et al., 2007; Melo et al., 2009; Veech & Crist, 2007). Although not identical, the environmental predictors (e.g., temperature range/seasonality for hermit thrushes vs. topographic heterogeneity and variation in transpiration rates for avian beta diversity) are highly correlated in the mountainous areas of western North America (Goulden et al., 2012; Roche et al., 2020), thus supporting a potential role for environmental heterogeneity in shaping patterns of avian diversity in western North America.

Higher turnover of putatively adaptive alleles in the west is concordant with high morphological diversity in fitness‐related traits (Dellinger et al., 2020). Although this needs to be tested directly, it supports the idea that environmental variation related to temperature may be important for divergence in traits related to thermal tolerance throughout the western range of hermit thrushes. Similar genotype–temperature correlations have been associated with ecologically relevant physiological traits including heat stress in willow flycatchers (Ruegg et al., 2018), providing insight into possible mechanisms underlying temperature‐related climate adaptations. Additional heritable traits associated with temperature and thermoregulation in birds include melanin‐based pigmentation (Galván et al., 2018; Romano et al., 2019), bill size (Romano et al., 2021; Tattersall et al., 2017), and wing length (Romano et al., 2021). Western subspecies of hermit thrushes vary extensively in both plumage coloration and morphometrics (Aldrich, 1968). Thus, we propose that future research include a genome‐wide association study (GWAS), which is needed to directly test for correlations between high turnover of putatively adaptive alleles and specific fitness‐related phenotypic traits in western populations of hermit thrushes.

Although in this study we focus on putative adaptive variation, we do not discount the role of historical processes in structuring genetic variation across the breeding range. For example, some of the observed patterns could also be explained by neutral or adaptive divergence in the past that is currently maintained by contemporary barriers to gene flow (Hewitt, 2004; Shafer et al., 2010; Weir & Schluter, 2004). Our genoscape map corroborates genomic splits between the three main groups of subspecies (Figure 1, Figure S1). A previous study of hermit thrushes dated the major split between Western Lowland and Northern groups to the Last Glacial Maximum (Alvarado et al., 2014) and other studies of avian species occupying the same range suggest the Western Mountain group likely split from the Western Lowland group during the Pleistocene as well (Dohms et al., 2017; van Els et al., 2012; Weir & Schluter, 2004). However, our genoscape analyses uncovered two additional genomic breaks along the Pacific coast within the range of the Western Lowland subspecies group, which has the most subspecific variation (Dellinger et al., 2020). An updated demographic analysis of hermit thrushes is necessary to tease apart historical vs. contemporary and neutral vs. adaptive processes (Gutenkunst et al., 2009; Liu & Fu, 2015). However, continuously distributed divergent populations that resulted from secondary contact provide an opportunity to investigate how historical differences can be maintained via contemporary barriers to gene flow and/or recurrent selection (Barton & Gale, 1993; Garrick et al., 2019; Jones et al., 2013; Rellstab et al., 2015). Our investigation at the hybrid zone (Section 4.2) between the Western Lowland and Northern subspecies groups (i.e., representing the secondary contact zone between the western and eastern lineages) reveals how deep historical divisions can continue to be sculpted by the current environment.

### 4.2 Temperature associations across a local hybrid zone

Since the pattern at the macrogeographic scale represents an aggregate of ecological and evolutionary processes occurring at the microgeographic scale (Hoban et al., 2016; Rellstab et al., 2015), we investigate whether the rangewide environmental predictors and associated candidate loci are corroborated at the local scale. Our study indicates that the rangewide environmental predictors and the associated loci vary in their importance within the hybrid zone in British Columbia. Specifically, rangewide LFMM analyses identified SNPs that are strongly associated with the top uncorrelated environmental variables, which are all temperature related (i.e., mean diurnal temperature range (BIO2), temperature seasonality (BIO4), and maximum temperature of the warmest month (BIO5)). When mapped across the hybrid zone, however, the rangewide top candidate loci associated with diurnal temperature range (BIO2) show no variation in allele frequency, whereas the loci associated with temperature seasonality and maximum temperature of the warmest month (BIO4 and BIO5, respectively) exhibit clinal changes in allele frequency at the microgeographic scale (Figures 3 and 4). Only the top two candidate loci associated with temperature seasonality (BIO4) track the temperature gradient across the cline, suggesting a temperature‐associated barrier to gene flow and/or ecological selection (Kirk & Freeland, 2011; Rundle & Nosil, 2005; Vines et al., 2016). Notably, these are the two candidate variants identified as important at both scales. Although we may find more loci that are top candidates at both scales if we searched within all 27 environmental variables, we highlight these loci as it was mean diurnal temperature range (BIO2), temperature seasonality (BIO4), and maximum temperature of the warmest month (BIO5) that surfaced as the most important uncorrelated predictors at the rangewide scale.

Within the genome, the candidate loci we narrowed down as being important at both the macrogeographic scale rangewide and microgeographic scale of the hybrid zone fall near the prolactin gene *PRL* (Wilkanowska et al., 2014). Prolactin is a hormone that, in birds, has been linked to environmental conditions, stress, and reproduction (Angelier et al., 2016). Environmental stressors that affect avian prolactin levels include heat (Dawson & Sharp, 2010; Gahali et al., 2001; Rozenboim et al., 2004), drought (Delehanty et al., 1997), and food availability (Koch et al., 2004; Riechert et al., 2014). We do not want to overinflate the relevance of prolactin in this study system. Importantly, confirmation of allele frequency shifts of the prolactin gene itself as well as functional tests confirming its biological significance in hermit thrushes would be necessary before making any inferences. Nevertheless, as the loci situated near the prolactin gene were the only candidates identified as highly ranked at both spatial scales, it provides a possible avenue for future molecular research in this system, especially given its association with heat stress as well as onset of reproduction (Angelier et al., 2016; Dawson & Sharp, 2010; Gahali et al., 2001; Rozenboim et al., 2004).

The clines for the top two candidate loci associated with temperature seasonality (BIO4) are shifted westward compared to the extremely steep cline observed for the full genomic dataset (Figure 4). Similarly, temperature shifts along the hybrid zone transect in British Columbia are also shifted westward in comparison to a major habitat shift (Hamann & Wang, 2006), which appears to coincide more closely with the major genomic break. This suggests that temperature‐related barriers to gene flow and/or ecological selection (Barton & Hewitt, 1985; Rice et al., 2011; Rundle & Nosil, 2005) may be operating separately from other ecological drivers such as habitat (Vines et al., 2016). To further explore the differences in cline shape, detailed investigations of exogenous factors (e.g., temperature, habitat, and behavioral differences) as well as potential endogenous factors (e.g., genetic incompatibility and hybrid sterility) are warranted (Bierne et al., 2011; Carling & Brumfield, 2008; Fitzpatrick & Shaffer, 2004).

It is also informative that the top rangewide candidate loci associated with mean diurnal temperature range (BIO2) do not show any clinal variation across the hybrid zone. This confirms that not all of the candidate loci associated with the top rangewide environmental predictors are expected to show a clinal pattern at a local scale (Poncet et al., 2010; Rellstab et al., 2015). Instead, patterns of concordance may vary predictably across hybrid zones in this system, and some of this variation may be detectable at the broad scale. Rangewide, Figure 2b indicates that mean diurnal temperature range (BIO2) differentiates populations across PC2 (and thus likely plays a stronger role across latitude). In contrast, temperature seasonality (BIO4) differentiates populations across PC1 (and thus likely plays a stronger role across longitude, as seen at this west to east‐oriented hybrid zone in British Columbia) (Figure 2b). Although more sampling is required to replicate these analyses, we propose that future work investigate other microgeographic sites to identify how patterns compare to British Columbia (Gugger et al., 2021). Another potential west to east‐oriented contact zone between the West‐South and West‐Interior clusters in the Sierra Nevada mountain range of California could serve as a replicate to investigate parallel drivers of divergence (i.e., temperature seasonality, or BIO4) and top‐associated candidate loci (i.e., candidate variants near the *PRL* gene) across a longitudinal gradient. Conversely, a potential north to south‐oriented contact zone in the Pacific Northwest between clusters in California and British Columbia could test the prediction that other temperature variables such as mean diurnal temperature range (BIO2) and its associated candidate loci are important across latitudinal gradients in the western region.

### 4.3 Implications for conservation and management

Informed conservation efforts on the breeding grounds of wildlife should account for geographic variation in the adaptive potential of populations (Funk et al., 2019). High levels of putatively adaptive genetic variation coinciding with large phenotypic and behavioral differences among western ecotypes of hermit thrushes may reflect high potential for rapid evolutionary change (Morgan et al., 2020; Thomassen et al., 2011), as they likely contain the raw material for ongoing natural selection to act upon (Lawson & Petren, 2017). Also, because genotype–environment associations include temperature regimes as the strongest predictors of genomic variation, this species may be especially sensitive to climate change. Under future climate scenarios that indicate increased average surface temperatures of 1–4°C (IPCC, 2018), the aggregate of western populations of hermit thrushes as a whole could provide a reservoir of standing genomic and phenotypic variation to potentially buffer against these changes. Thus, local extinctions or population reductions throughout the west may have greater impact on loss of genetic diversity and adaptive potential of hermit thrushes than formerly realized. Preserving such reservoirs of adaptive potential is especially relevant because while some species or populations may shift their ranges (MacLean & Beissinger, 2017; Pacifici et al., 2015), others may be required to adjust to climate change *in situ* via plastic or genetic responses (Bay et al., 2018; Fitzpatrick & Keller, 2015; Ruegg et al., 2018; Williams et al., 2008).

Consideration of genotype–environment associations as well as degree of threat (Hoekstra et al., 2005; Tulloch et al., 2015) to populations could inform management strategies for breeding ground populations. One group in particular that may be especially vulnerable is the West‐South cluster occupying coastal California (Figures 1 and 2). This ecotype, traditionally identified as the Monterey hermit thrush (*C.g. slevini*), may potentially be considered an evolutionary significant unit (Funk et al., 2012, 2019; Moritz, 1994), based on its unique gene–environment correlations and potential adaptive differences (Nelson et al., 2016, 2021). Furthermore, this group is currently experiencing heightened threat levels due to fires (Bock & Lynch, 1970; Goss et al., 2020; Nelson et al., 2021; Taillie et al., 2018) and ongoing habitat destruction (Kalinowski & Johnson, 2010; Pidgeon et al., 2007). Greater sampling coverage in the Pacific Northwest would inform our understanding of the northern limits of this ecotype's geographic range and the extent of gene flow with other ecotypes. Addressing sampling gaps within the expansive area occupied by the East‐Taiga cluster (Figures 1 and 2) is also worthwhile, even though it is unlikely that hidden genetic variation is harbored within central Canada. This group occupies areas of high latitude which are predicted to be heavily impacted by climate change (Bateman et al., 2020); therefore anticipating its response to future environmental change will be important (DesGranges & Morneau, 2010; Stralberg et al., 2019).

## 5 CONCLUSION

Our study reveals how investigations at the microgeographic and macrogeographic scales can complement one another. We find higher turnover of putatively adaptive alleles in western North America compared to eastern and boreal regions, and this could be due to high environmental heterogeneity in the west. The main environmental predictors of the pattern uncovered at the macrogeographic scale are all related to temperature; however, these vary in importance at the microgeographic scale. Only the candidate loci associated with temperature seasonality (BIO4) stand out as highly ranked at both scales. At the hybrid zone in British Columbia, we also find concordance of these particular candidate loci with a temperature‐related gradient, suggesting potential temperature‐driven barriers to gene flow and/or a role for temperature‐related ecological selection in maintaining putative local adaptation. Thus, we confirm that the hybrid zone analysis can be used to validate some aspects of the broadscale analysis and can expose possible ecological and evolutionary mechanisms underlying putative climate adaptation detected at the broad scale. From a conservation perspective, high levels of putatively adaptive genetic variation coinciding with large phenotypic and behavioral differences among distinct hermit thrush ecotypes throughout the west may reflect high adaptive potential, which is becoming increasingly important as species may be required to adapt *in situ* to rapid environmental change. As a sense of urgency for conservation action climbs, there are enhanced opportunities to learn about the potential impacts of climate change from species, such as the hermit thrush, for which temperature is an important predictor of putative local adaptation.

## CONFLICT OF INTEREST

The authors declare no conflict of interest.

## Supporting information


Supplementary Material

## Data Availability

Data for this study are available at the Dryad Digital Repository: https://doi.org/10.5061/dryad.xpnvx0kjh.
