# Supplementary material for: Genotype–environment associations across spatial scales reveal the importance of putative adaptive genetic variation in divergence
Source: Evol Appl. 2022 Aug 24;15(9):1390–407. doi: 10.1111/eva.13444 (PMC9488676; doi:10.1111/eva.13444)
Supplement: Supplementary file 1 — Supplementary Material [file EVA-15-1390-s001.pdf]

**Supporting Information**

# GENOTYPE-ENVIRONMENT ASSOCIATIONS ACROSS SPATIAL SCALES REVEAL THE IMPORTANCE OF PUTATIVE ADAPTIVE GENETIC VARIATION IN DIVERGENCE

Allison H. Alvarado[1*], Christen M. Bossu[2,6], Ryan J. Harrigan[2], Rachael A. Bay[3], Allison R. P. Nelson[4], Thomas B. Smith[2,5], and Kristen C. Ruegg[6]

[1] *Biology Department, California State University Channel Islands, Camarillo, CA, USA*

[2] *Center for Tropical Research, Institute of Environment and Sustainability, University of California, Los Angeles, CA, USA*

[3] *Department of Evolution and Ecology, University of California, Davis, CA, USA*

[4] *Gold Country Avian Studies, 14401 Pierite Rd, Nevada City, CA, USA*

[5] *Department of Ecology and Evolutionary Biology, University of California, Los Angeles, CA, USA*

[6] *Department of Biology, Colorado State University, Fort Collins, CO, USA*

*Corresponding Author: Allison H. Alvarado, *Biology Department, California State University Channel Islands, Camarillo, CA 93012, USA; Email address: allison.alvarado@csuci.edu*

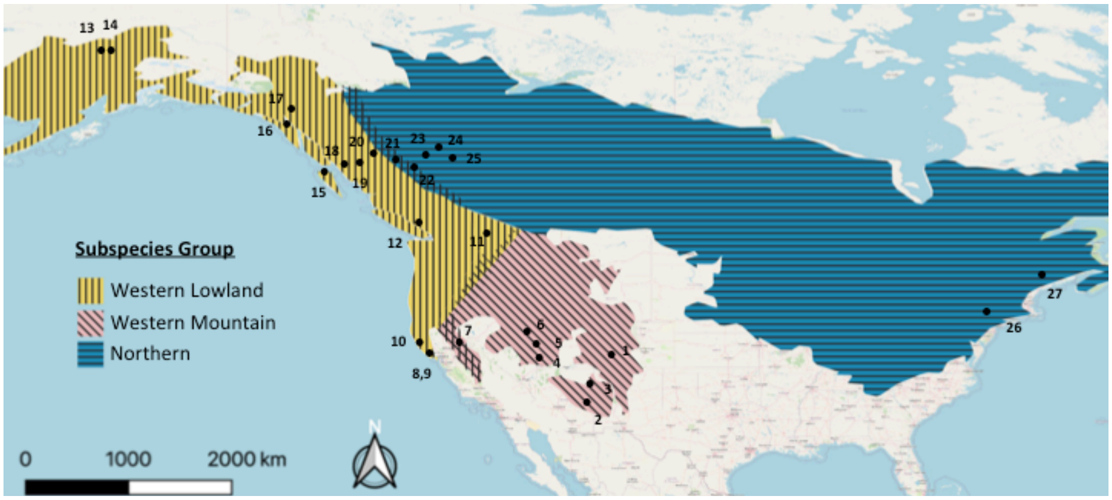


**Figure S1.** The hermit thrush (*Catharus guttatus*) has historically been divided into three main geographically concordant groups of subspecies, based on variation in size, wing and bill length and shape, and plumage coloration. Pyle (1997) and Dellinger et al. (2020) label these groups as Western Lowland, Western Mountain, and Northern (based primarily on Phillips 1961, 1991). Hermit thrushes are phenotypically variable within each group, especially throughout the western portion of the range, and each group is comprised of multiple subspecies (Phillips 1991). The majority of subspecies (10 out of 12) occur within the Western Lowland and Western Mountain groups, while the remaining two subspecies occur within the Northern group (Aldrich 1968, Dellinger et al. 2020). The Western Lowland group spans the length of the west coast from Alaska through California and is comprised of seven subspecies varying from medium-sized and dark to the smallest, very pale birds. The Western Mountain group spans from California's Sierra Nevada range to the United States' Rocky Mountains and into the mountains of the desert Southwest. This group, characterized by the largest and palest birds, is comprised of three subspecies. The Northern group stretches from the boreal forest of central Alaska eastward across Canada to the east coast from Newfoundland to Virginia. This group, comprised of two subspecies, is medium-sized and has rufous coloration.

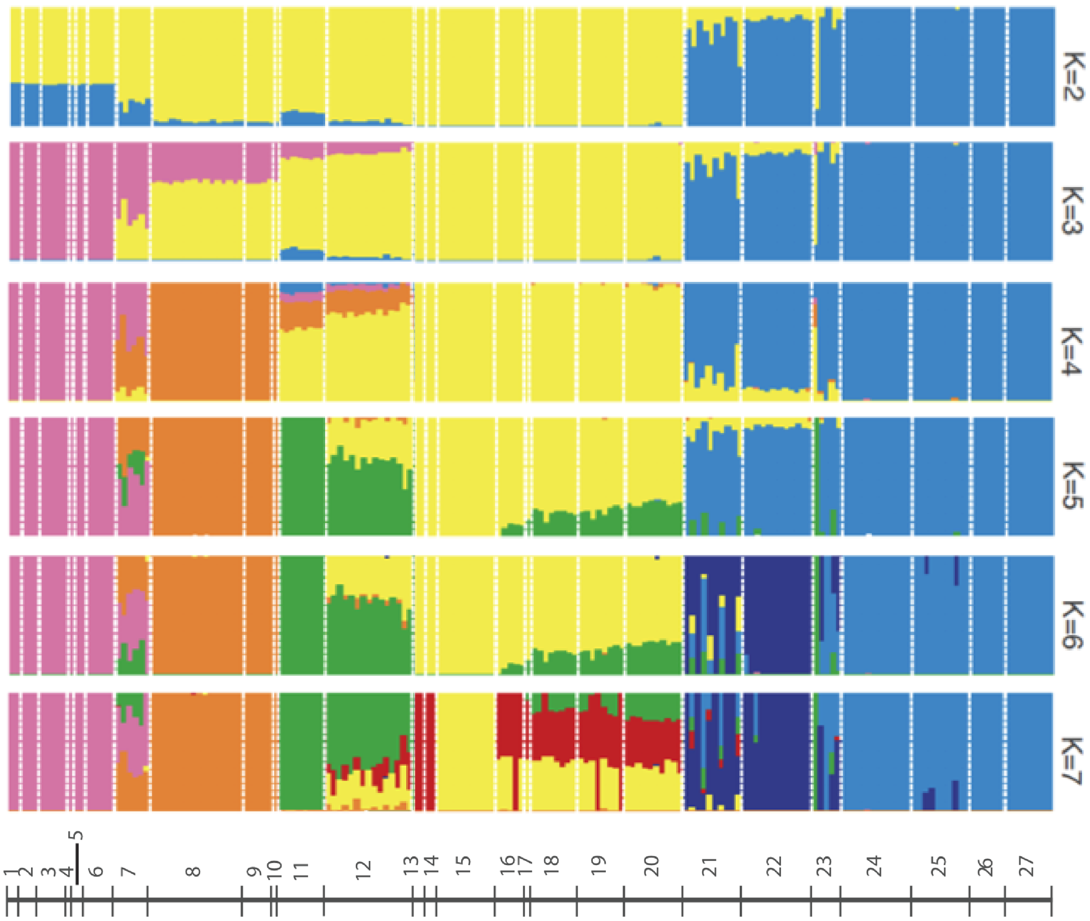


**Figure S2.** Ancestry results from the admixture analyses for genetic clusters, showing K ranging from 2:7. We visualized each run and estimated the cross-validation error to determine an optimal K=5. There is a clear split between east versus west (K=2). The West-North group in the west is partitioned into the additional genetic groups with each addition of K in the following order (K=3, West-Interior; K=4, West-South; K=5, West-Central). At K=6, individuals at the hybrid zone in central British Columbia are partitioned. At K=7, individuals from central Alaska are partitioned.

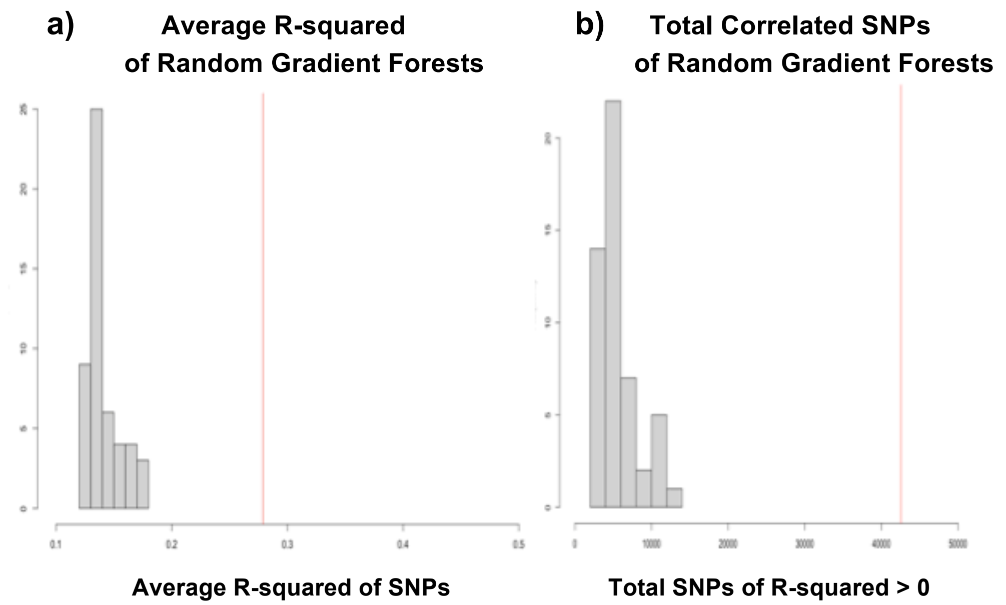


**Figure S3.** Observed results (red lines) of gradient forest compared to runs with randomized matches (grey histograms). The observed responses were significantly greater in **(a)** correlation value and **(b)** total number of correlated SNPs compared to gradient forest runs where genomic signatures and predictors were randomized with respect to one another (p<0.01).

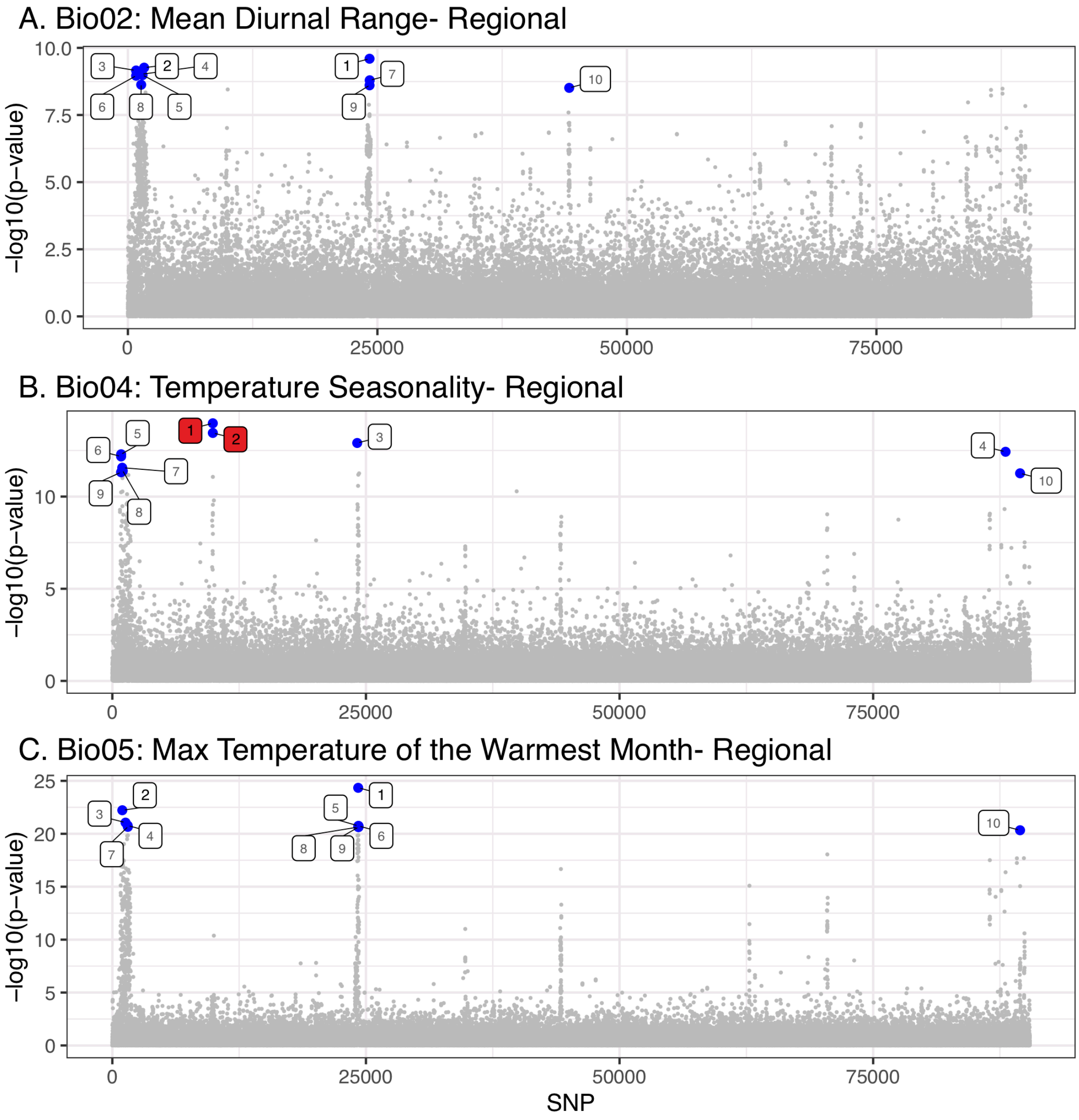


**Figure S4.** Association of candidate loci at macrogeographic scale identified using LFMM with top ranking uncorrelated environmental variables found in gradient forest. LFMM was run on the macrogeographic (rangewide) sampling sites, excluding six hybrid zone sites. The top 10 candidate loci for each environmental variable are labeled. Red labeling corresponds to two candidate loci identified at the macrogeographic scale that are also candidate loci at the microgeographic scale (see Figure S5).

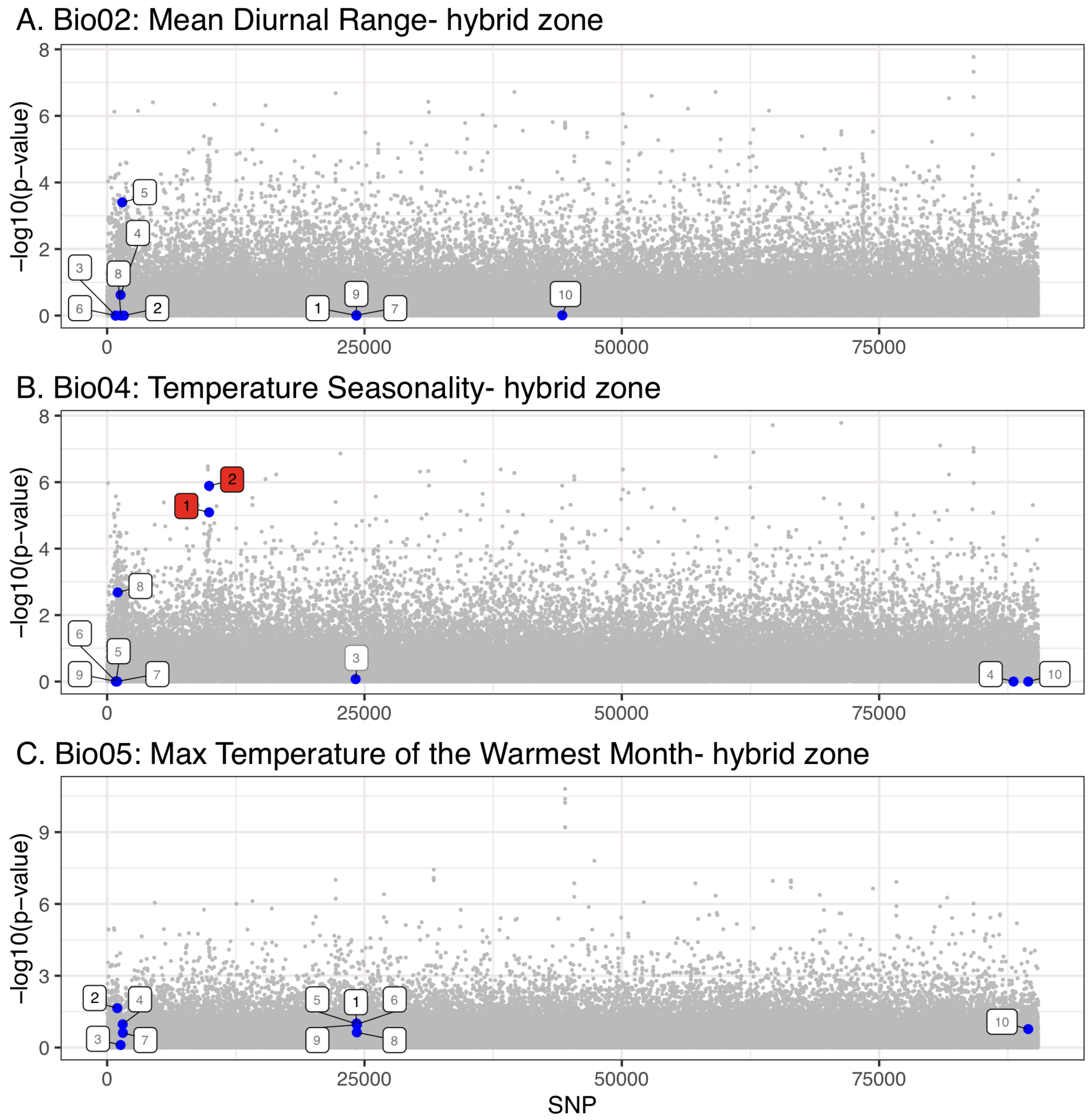


**Figure S5.** Association of candidate loci at microgeographic scale identified using LFMM with top ranking uncorrelated environmental variables found in gradient forest. LFMM was run within the microgeographic (hybrid zone) sampling sites. The top 10 candidate loci for the top ranking uncorrelated environmental variables identified at the regional scale are labeled (see Figure S4). Red labeling corresponds to two candidate loci identified at the microgeographic scale that are also candidate loci at the macrogeographic scale.

**Table S1.** Environmental variables used in the gradient forest analysis, ordered by $R^2$ weighted importance rank. The highest environmental predictors of the full gradient forest model are BIO2, BIO4, and BIO7, whereas the highest uncorrelated environmental predictors are BIO2, BIO4, and BIO5.

| Variable | Description | Source/ Format | $R^2$ Weighted Importance Rank | Accuracy Importance Rank |
|---|---|---|---|---|
| BIO2 | Mean diurnal range (Mean of monthly (max temp – min temp)) | WorldClim | 1 | 2 |
| BIO4 | Temperature seasonality (standard deviation x100) | WorldClim | 2 | 3 |
| BIO7 | Annual temperature range (BIO5-BIO6) | WorldClim | 3 | 4 |
| BIO9 | Mean temperature of driest quarter | WorldClim | 4 | 1 |
| Long | Mean longitude of population | WGS84 | 5 | 6 |
| BIO19 | Precipitation of coldest quarter | WorldClim | 6 | 5 |
| BIO5 | Max temperature of warmest month | WorldClim | 7 | 7 |
| BIO16 | Precipitation of wettest quarter | WorldClim | 8 | 8 |
| BIO13 | Precipitation of wettest month | WorldClim | 9 | 12 |
| BIO10 | Mean temperature of warmest quarter | WorldClim | 10 | 10 |
| BIO12 | Annual precipitation | WorldClim | 11 | 16 |
| BIO6 | Min temperature of coldest month | WorldClim | 12 | 17 |
| QuickScat | Surface moisture characteristics | NASA Scatter | 13 | 11 |
| Lat | Mean latitude of population | WGS84 | 14 | 13 |
| SRTM | Elevation | WorldClim | 15 | 9 |
| BIO8 | Mean temperature of wettest quarter | WorldClim | 16 | 15 |
| BIO15 | Precipitation seasonality (Coeff. of Variation) | WorldClim | 14 | 15 |
| BIO17 | Precipitation of driest quarter | WorldClim | 18 | 19 |
| BIO14 | Precipitation of driest month | WorldClim | 19 | 21 |
| BIO18 | Precipitation of warmest quarter | WorldClim | 20 | 22 |
| BIO11 | Mean temperature of coldest quarter | WorldClim | 21 | 23 |
| NDVIstdv | Vegetation indices | MODIS | 22 | 18 |
| BIO3 | Isothermality (BIO2/BIO7) (x100) | WorldClim | 23 | 24 |
| NDVImax | Vegetation indices | MODIS | 24 | 20 |
| BIO1 | Annual mean temperature | WorldClim | 27 | 25 |
| HII | Human influence index | WCS | 26 | 26 |
| TreeCover | Tree cover | MODIS/ LandSat | 27 | 27 |

**Table S2.** $F_{ST}$ values for pairwise comparisons of the19 populations used in rangewide analyses. Grey shading indicates the comparisons between divergent eastern vs western lineages.

| | Arizona | Utah | Yosemite, CA | Coastal CA_1 | Coastal CA_1 | HaidaGwaii, BC | Alaska_SE | PrinceRupert, B | Exstew, BC | Kispiox, BC | Whistler, BC | Golden, BC | Maxan, BC | FortFraser, BC | MacKenzie, BC | Hudson's Hope | Swan Lake, BC | Pennsylvania | Maine |
|---|---|---|---|---|---|---|---|---|---|---|---|---|---|---|---|---|---|---|---|
| Arizona | 0 | 0.0069 | 0.0342 | 0.0933 | 0.1109 | 0.135 | 0.1299 | 0.1214 | 0.1209 | 0.1157 | 0.0964 | 0.0903 | 0.1094 | 0.1269 | 0.104 | 0.1452 | 0.1444 | 0.1506 | 0.1515 |
| Utah | 0.0069 | 0 | 0.0247 | 0.0841 | 0.1001 | 0.1252 | 0.1198 | 0.1122 | 0.1111 | 0.1063 | 0.0871 | 0.0809 | 0.0994 | 0.1164 | 0.0928 | 0.1352 | 0.1344 | 0.1392 | 0.141 |
| Yosemite, CA | 0.0342 | 0.0247 | 0 | 0.0274 | 0.0388 | 0.0675 | 0.0586 | 0.0533 | 0.0529 | 0.0499 | 0.0344 | 0.0291 | 0.0754 | 0.0982 | 0.0639 | 0.1193 | 0.1181 | 0.1212 | 0.1236 |
| Coastal CA_1 | 0.0933 | 0.0841 | 0.0274 | 0 | 0.0098 | 0.0544 | 0.0484 | 0.0426 | 0.0425 | 0.0396 | 0.0288 | 0.0289 | 0.0928 | 0.1167 | 0.0844 | 0.1388 | 0.1372 | 0.1409 | 0.1431 |
| Coastal CA_1 | 0.1109 | 0.1001 | 0.0388 | 0.0098 | 0 | 0.066 | 0.0582 | 0.0542 | 0.0527 | 0.0505 | 0.0381 | 0.0393 | 0.1056 | 0.1318 | 0.0964 | 0.1575 | 0.1572 | 0.162 | 0.1648 |
| HaidaGwaii, BC | **0.135** | 0.1252 | 0.0675 | 0.0544 | 0.066 | 0 | 0.0063 | 0.0055 | 0.0066 | 0.0073 | 0.0161 | 0.028 | 0.1159 | 0.1481 | 0.1097 | 0.1759 | 0.1737 | 0.1779 | 0.181 |
| Alaska_SE | 0.1299 | 0.1198 | 0.0586 | 0.0484 | 0.0582 | 0.0063 | 0 | 0.0014 | 0.0025 | 0.0024 | 0.0092 | 0.0195 | 0.1085 | 0.1423 | 0.1002 | 0.173 | 0.1709 | 0.1751 | 0.1788 |
| PrinceRupert, B | 0.1214 | 0.1122 | 0.0533 | 0.0426 | 0.0542 | 0.0055 | 0.0014 | 0 | 4.00E-04 | 3.00E-04 | 0.0067 | 0.0155 | 0.1021 | 0.1345 | 0.0941 | 0.1623 | 0.1602 | 0.164 | 0.1676 |
| Exstew, BC | 0.1209 | 0.1111 | 0.0529 | 0.0425 | 0.0527 | 0.0066 | 0.0025 | 0.0004 | 0 | 6.00E-04 | 0.0061 | 0.0156 | 0.1009 | 0.1332 | 0.0932 | 0.1608 | 0.1589 | 0.1628 | 0.166 |
| Kispiox, BC | 0.1157 | 0.1063 | 0.0499 | 0.0396 | 0.0505 | 0.0073 | 0.0024 | 0.0003 | 6.00E-04 | 0 | 0.0054 | 0.0129 | 0.0948 | 0.1261 | 0.0868 | 0.1523 | 0.1502 | 0.1538 | 0.1573 |
| Whistler, BC | 0.0964 | 0.0871 | 0.0344 | 0.0288 | 0.0381 | 0.0161 | 0.0092 | 0.0067 | 0.0061 | 0.0054 | 0 | 0.0044 | 0.0822 | 0.1106 | 0.0727 | 0.1338 | 0.1319 | 0.135 | 0.1378 |
| Golden, BC | 0.0903 | 0.0809 | 0.0291 | 0.0289 | 0.0393 | 0.028 | 0.0195 | 0.0155 | 0.0156 | 0.0129 | 0.0044 | 0 | 0.0725 | 0.1 | 0.0613 | 0.1233 | 0.1212 | 0.1245 | 0.1272 |
| Maxan, BC | 0.1094 | 0.0994 | 0.0754 | 0.0928 | 0.1056 | 0.1159 | 0.1085 | 0.1021 | 0.1009 | 0.0948 | 0.0822 | 0.0725 | 0 | 0.0032 | 0.0015 | 0.0106 | 0.0105 | 0.013 | 0.0139 |
| FortFraser, BC | 0.1269 | 0.1164 | 0.0982 | 0.1167 | 0.1318 | 0.1481 | 0.1423 | 0.1345 | 0.1332 | 0.1261 | 0.1106 | 0.1 | 0.0032 | 0 | 0.0069 | 0.0046 | 0.004 | 0.0059 | 0.0078 |
| MacKenzie, BC | 0.104 | 0.0928 | 0.0639 | 0.0844 | 0.0964 | 0.1097 | 0.1002 | 0.0941 | 0.0932 | 0.0868 | 0.0727 | 0.0613 | 0.0015 | 0.0069 | 0 | 0.0149 | 0.0139 | 0.0153 | 0.0182 |
| Hudson's Hope | 0.1452 | 0.1352 | 0.1193 | 0.1388 | 0.1575 | 0.1759 | 0.173 | 0.1623 | 0.1608 | 0.1523 | 0.1338 | 0.1233 | 0.0106 | 0.0046 | 0.0149 | 0 | 0 | 0.0015 | 0.0024 |
| Swan Lake, BC | 0.1444 | 0.1344 | 0.1181 | 0.1372 | 0.1572 | 0.1737 | 0.1709 | 0.1602 | 0.1589 | 0.1502 | 0.1319 | 0.1212 | 0.0105 | 0.004 | 0.0139 | 0 | 0 | 6.00E-04 | 0.0021 |
| Pennsylvania | 0.1506 | 0.1392 | 0.1212 | 0.1409 | 0.162 | 0.1779 | 0.1751 | 0.164 | 0.1628 | 0.1538 | 0.135 | 0.1245 | 0.013 | 0.0059 | 0.0153 | 0.0015 | 6.00E-04 | 0 | 5.00E-04 |
| Maine | 0.1515 | 0.141 | 0.1236 | 0.1431 | 0.1648 | 0.181 | 0.1788 | 0.1676 | 0.166 | 0.1573 | 0.1378 | 0.1272 | 0.0139 | 0.0078 | 0.0182 | 0.0024 | 0.0021 | 5.00E-04 | 0 |

**Table S3.** Partial Mantel tests reveal contrasting patterns for eastern and western lineages when analyzed separately. The western lineage shows significant patterns of isolation by environment (r = .414, p = .01) and isolation by distance (r = .657, p = .001). In contrast, the eastern lineage shows no evidence of isolation by environment (r = .189, p = .176) or isolation by distance (r = .181, p = .114).

| | All populations | West only | East only |
|---|---|---|---|
| Genetic vs Geographic distance | r = .315<br>P = .002** | r = .657<br>P = .001** | r = .181<br>P = .114(ns) |
| Genetic vs Environmental distance | r = .756<br>P = .001** | r = .414<br>P = .01* | r = .189<br>P = .176 (ns) |

**Table S4.** Pearson's Correlation Coefficients for pairwise comparisons of the 27 environmental variables used in the gradient forest.

| | Lat_mea | Long_m | BIO1 | BIO2 | BIO3 | BIO4 | BIO5 | BIO6 | BIO7 | BIO8 | BIO9 | BIO10 | BIO11 | BIO12 | BIO13 | BIO14 | BIO15 | BIO16 | BIO17 | BIO18 | BIO19 | NDVIma | NDVIstd | QuickSc | SRTM | TreeCov | HII |
|---|---|---|---|---|---|---|---|---|---|---|---|---|---|---|---|---|---|---|---|---|---|---|---|---|---|---|---|
| Lat_mea | 1 | -0.36 | -0.48 | -0.78 | -0.82 | 0.048 | -0.81 | -0.1 | -0.31 | -0.38 | -0.26 | -0.58 | -0.33 | 0.634 | 0.53 | 0.681 | -0.35 | 0.559 | 0.684 | 0.679 | 0.427 | 0.126 | 0.473 | -0.24 | -0.43 | 0.041 | 0.308 |
| Long-me | -0.36 | 1 | 0.007 | 0.441 | -0.17 | 0.727 | 0.638 | -0.5 | 0.751 | 0.198 | -0.7 | 0.606 | -0.39 | -0.39 | -0.58 | 0.037 | -0.55 | -0.58 | 0.021 | -0.1 | -0.57 | 0.085 | 0.351 | 0.626 | -0.09 | 0.072 | 0.156 |
| BIO1 | -0.48 | 0.007 | 1 | 0.023 | 0.653 | -0.57 | 0.6 | 0.789 | -0.4 | 0.347 | 0.56 | 0.743 | 0.894 | 0.009 | 0.169 | -0.33 | 0.634 | 0.156 | -0.32 | -0.37 | 0.303 | 0.591 | -0.28 | 0.225 | -0.48 | 0.65 | 0.106 |
| BIO2 | -0.78 | 0.441 | 0.023 | 1 | 0.56 | 0.391 | 0.701 | -0.43 | 0.724 | 0.433 | -0.16 | 0.371 | -0.18 | -0.75 | -0.71 | -0.61 | 0.091 | -0.74 | -0.62 | -0.45 | -0.75 | -0.22 | -0.12 | 0.097 | 0.623 | -0.33 | -0.37 |
| BIO3 | -0.82 | -0.17 | 0.653 | 0.56 | 1 | -0.51 | 0.582 | 0.477 | -0.13 | 0.385 | 0.682 | 0.401 | 0.677 | -0.39 | -0.15 | -0.72 | 0.774 | -0.19 | -0.72 | -0.62 | -0.07 | -0.01 | -0.63 | -0.06 | 0.31 | 0.042 | -0.33 |
| BIO4 | 0.048 | 0.727 | -0.57 | 0.391 | -0.51 | 1 | 0.217 | -0.92 | 0.915 | 0.049 | -0.94 | 0.126 | -0.87 | -0.47 | -0.67 | 0.04 | -0.69 | -0.67 | 0.025 | 0.048 | -0.77 | -0.09 | 0.548 | 0.358 | 0.174 | -0.31 | -0.05 |
| BIO5 | -0.81 | 0.638 | 0.6 | 0.701 | 0.582 | 0.217 | 1 | 0.016 | 0.478 | 0.511 | -0.06 | 0.915 | 0.247 | -0.61 | -0.56 | -0.57 | 0.23 | -0.58 | -0.57 | -0.56 | -0.49 | 0.32 | -0.05 | 0.484 | -0.02 | 0.245 | -0.09 |
| BIO6 | -0.1 | -0.5 | 0.789 | -0.43 | 0.477 | -0.92 | 0.016 | 1 | -0.87 | 0.049 | 0.841 | 0.203 | 0.961 | 0.435 | 0.614 | -0.05 | 0.672 | 0.614 | -0.03 | -0.12 | 0.751 | 0.355 | -0.43 | -0.09 | -0.45 | 0.543 | 0.164 |
| BIO7 | -0.31 | 0.751 | -0.4 | 0.724 | -0.13 | 0.915 | 0.478 | -0.87 | 1 | 0.208 | -0.77 | 0.273 | -0.72 | -0.68 | -0.82 | -0.24 | -0.48 | -0.83 | -0.25 | -0.17 | -0.9 | -0.15 | 0.351 | 0.318 | 0.383 | -0.36 | -0.19 |
| BIO8 | -0.38 | 0.198 | 0.347 | 0.433 | 0.385 | 0.049 | 0.511 | 0.049 | 0.208 | 1 | -0.09 | 0.44 | 0.179 | -0.24 | -0.19 | -0.25 | 0.114 | -0.23 | -0.24 | -0.03 | -0.32 | 0.292 | 0.007 | 0.157 | -0.01 | -0.11 | 0.039 |
| BIO9 | -0.26 | -0.7 | 0.56 | -0.16 | 0.682 | -0.94 | -0.06 | 0.841 | -0.77 | -0.09 | 1 | -0.07 | 0.849 | 0.222 | 0.462 | -0.31 | 0.813 | 0.457 | -0.29 | -0.31 | 0.596 | -0.02 | -0.65 | -0.35 | 0.055 | 0.234 | -0.17 |
| BIO10 | -0.58 | 0.606 | 0.743 | 0.371 | 0.401 | 0.126 | 0.915 | 0.203 | 0.273 | 0.44 | -0.07 | 1 | 0.37 | -0.39 | -0.36 | -0.39 | 0.213 | -0.37 | -0.38 | -0.44 | -0.26 | 0.608 | 0.088 | 0.556 | -0.4 | 0.526 | 0.082 |
| BIO11 | -0.33 | -0.39 | 0.894 | -0.18 | 0.677 | -0.87 | 0.247 | 0.961 | -0.72 | 0.179 | 0.849 | 0.37 | 1 | 0.245 | 0.452 | -0.24 | 0.755 | 0.442 | -0.22 | -0.26 | 0.586 | 0.378 | -0.47 | -0.06 | -0.35 | 0.539 | 0.078 |
| BIO12 | 0.634 | -0.39 | 0.009 | -0.75 | -0.39 | -0.47 | -0.61 | 0.435 | -0.68 | -0.24 | 0.222 | -0.39 | 0.245 | 1 | 0.947 | 0.837 | -0.16 | 0.96 | 0.851 | 0.787 | 0.891 | 0.186 | 0.276 | -0.38 | -0.47 | 0.417 | 0.614 |
| BIO13 | 0.53 | -0.58 | 0.169 | -0.71 | -0.15 | -0.67 | -0.56 | 0.614 | -0.82 | -0.19 | 0.462 | -0.36 | 0.452 | 0.947 | 1 | 0.645 | 0.141 | 0.993 | 0.66 | 0.657 | 0.938 | 0.208 | 0.098 | -0.49 | -0.43 | 0.44 | 0.519 |
| BIO14 | 0.681 | 0.037 | -0.33 | -0.61 | -0.72 | 0.04 | -0.57 | -0.05 | -0.24 | -0.25 | -0.31 | -0.39 | -0.24 | 0.837 | 0.645 | 1 | -0.64 | 0.659 | 0.997 | 0.908 | 0.533 | 0.041 | 0.54 | -0.14 | -0.39 | 0.224 | 0.654 |
| BIO15 | -0.35 | -0.55 | 0.634 | 0.091 | 0.774 | -0.69 | 0.23 | 0.672 | -0.48 | 0.114 | 0.813 | 0.213 | 0.755 | -0.16 | 0.141 | -0.64 | 1 | 0.117 | -0.64 | -0.54 | 0.225 | 0.144 | -0.57 | -0.14 | -0.01 | 0.181 | -0.33 |
| BIO16 | 0.559 | -0.58 | 0.156 | -0.74 | -0.19 | -0.67 | -0.58 | 0.614 | -0.83 | -0.23 | 0.457 | -0.37 | 0.442 | 0.96 | 0.993 | 0.659 | 0.117 | 1 | 0.674 | 0.642 | 0.958 | 0.207 | 0.107 | -0.45 | -0.46 | 0.444 | 0.501 |
| BIO17 | 0.684 | 0.021 | -0.32 | -0.62 | -0.72 | 0.025 | -0.57 | -0.03 | -0.25 | -0.24 | -0.29 | -0.38 | -0.22 | 0.851 | 0.66 | 0.997 | -0.64 | 0.674 | 1 | 0.914 | 0.55 | 0.081 | 0.549 | -0.17 | -0.4 | 0.244 | 0.681 |
| BIO18 | 0.679 | -0.1 | -0.37 | -0.45 | -0.62 | 0.048 | -0.56 | -0.12 | -0.17 | -0.03 | -0.31 | -0.44 | -0.26 | 0.787 | 0.657 | 0.908 | -0.54 | 0.642 | 0.914 | 1 | 0.431 | 0.114 | 0.599 | -0.39 | -0.27 | 0.135 | 0.631 |
| BIO19 | 0.427 | -0.57 | 0.303 | -0.75 | -0.07 | -0.77 | -0.49 | 0.751 | -0.9 | -0.32 | 0.596 | -0.26 | 0.586 | 0.891 | 0.938 | 0.533 | 0.225 | 0.958 | 0.55 | 0.431 | 1 | 0.214 | -0.04 | -0.34 | -0.48 | 0.528 | 0.432 |
| NDVIma | 0.126 | 0.085 | 0.591 | -0.22 | -0.01 | -0.09 | 0.32 | 0.355 | -0.15 | 0.292 | -0.02 | 0.608 | 0.378 | 0.186 | 0.208 | 0.041 | 0.144 | 0.207 | 0.081 | 0.114 | 0.214 | 1 | 0.481 | 0.11 | -0.73 | 0.773 | 0.341 |
| NDVIstd | 0.473 | 0.351 | -0.28 | -0.12 | -0.63 | 0.548 | -0.05 | -0.43 | 0.351 | 0.007 | -0.65 | 0.088 | -0.47 | 0.276 | 0.098 | 0.54 | -0.57 | 0.107 | 0.549 | 0.599 | -0.04 | 0.481 | 1 | 0.104 | -0.34 | 0.381 | 0.416 |
| QuickSc | -0.24 | 0.626 | 0.225 | 0.097 | -0.06 | 0.358 | 0.484 | -0.09 | 0.318 | 0.157 | -0.35 | 0.556 | -0.06 | -0.38 | -0.49 | -0.14 | -0.14 | -0.45 | -0.17 | -0.39 | -0.34 | 0.11 | 0.104 | 1 | -0.28 | 0.111 | -0.04 |
| SRTM | -0.43 | -0.09 | -0.48 | 0.623 | 0.31 | 0.174 | -0.02 | -0.45 | 0.383 | -0.01 | 0.055 | -0.4 | -0.35 | -0.47 | -0.43 | -0.39 | -0.01 | -0.46 | -0.4 | -0.27 | -0.48 | -0.73 | -0.34 | -0.28 | 1 | -0.69 | -0.48 |
| TreeCov | 0.041 | 0.072 | 0.65 | -0.33 | 0.042 | -0.31 | 0.245 | 0.543 | -0.36 | -0.11 | 0.234 | 0.526 | 0.539 | 0.417 | 0.44 | 0.224 | 0.181 | 0.444 | 0.244 | 0.135 | 0.528 | 0.773 | 0.381 | 0.111 | -0.69 | 1 | 0.35 |
| HII | 0.308 | 0.156 | 0.106 | -0.37 | -0.33 | -0.05 | -0.09 | 0.164 | -0.19 | 0.039 | -0.17 | 0.082 | 0.078 | 0.614 | 0.519 | 0.654 | -0.33 | 0.501 | 0.681 | 0.631 | 0.432 | 0.341 | 0.416 | -0.04 | -0.48 | 0.35 | 1 |

## References


Aldrich, J. W. (1968). Population characteristics and nomenclature of the hermit thrush. *Proceedings of the United States National Museum, 124*(3637), 1-33.

Dellinger, R. P., Wood, B., Jones, P. W., & Donovan, T. M. (2020). Hermit Thrush (*Catharus guttatus*), version 1.0. In: A. F. Poole (Ed.), Birds of the World. Cornell Lab of Ornithology, Ithaca, NY, USA.

Phillips, A. R., Marshall, J., & Monson, G. (1964). The birds of Arizona. University of Arizona Press, Tucson, AZ.

Phillips, A. R. (1991). The known birds of North and Middle America: Part II. Allan R. Phillips, Denver, CO.

Pyle, P. (1997). Identification guide to North American birds: a compendium of information on identifying, ageing, and sexing "near-passerines" and passerines in the hand. Slate Creek Press, Bolinas, CA.
